# Supplementary material for: Cannabinoid-Inspired Inhibitors of the SARS-CoV-2 Coronavirus 2′-O-Methyltransferase (2′-O-MTase) Non-Structural Protein (Nsp10–16)
Source: Molecules. 2024 Oct 28;29(21):5081. doi: 10.3390/molecules29215081 (PMC11547505; doi:10.3390/molecules29215081)
Supplement: Supplementary file 1 [file molecules-29-05081-s001.zip › PROOF- SI- Cannabinoid-Inspired Inhibitors of the SARS-CoV-2-comments FINAL.pdf]

Supporting Information

Cannabinoid-Inspired Inhibitors of the SARS-CoV-2 Coronavirus 2′-*O*-Methyltransferase (2′-*O*-MTase) Non-Structural Protein (Nsp10–16)

Menny M. Benjamin [1], George S. Hanna [1], Cody F. Dickinson [1], Yeun-Mun Choo [2], Xiaojuan Wang [3], Jessica A. Downs-Bowen [4], Ramyani De [4], Tamara R. McBrayer [4], Raymond F. Schinazi [4], Sarah E. Nielson [5], Joan M. Hevel [5], Pankaj Pandey [6], Robert J. Doerksen [7], Jie Zhang [8], Zhiwei Ye [8], Danyelle M. Townsend [8], Scott Wyer [1], Lucas Bialousow [1] and Mark T. Hamann [1,9,*]

[1] Department of Drug Discovery & Biomedical Sciences, Medical University of South Carolina, 280 Calhoun St, Charleston, SC 29425, USA; benjamim@musc.edu (M.M.B.)
[2] Department of Chemistry, University of Malaya, Kuala Lumpur 50603, Malaysia
[3] Department of Pharmacy, Lanzhou University, Lanzhou 730000, China
[4] Center for ViroScience and Cure, Laboratory of Biochemical Pharmacology, Department of Pediatrics, Emory University School of Medicine and Children's Healthcare of Atlanta, 1760 Haygood Drive, HSRB-1, Atlanta, GA 30322, USA
[5] Department of Chemistry & Biochemistry, Logan, UT 84322, USA
[6] National Center for Natural Products Research, School of Pharmacy, University of Mississippi, University, MS 38677, USA
[7] Department of BioMolecular Sciences and Research Institute of Pharmaceutical Sciences, University of Mississippi, University, MS 38677, USA #
[8] Department of Cell and Molecular Pharmacology and Experimental Therapeutics, Medical University of South Carolina, 70 President St, DD410, Charleston, SC 29425, USA
[9] Department of Public Health Sciences, Medical University of South Carolina,135 Cannon St, Charleston, SC 29425, USA
* Correspondence: hamannm@musc.edu

Content **Page**

1. Experimental Procedures S2-S8
2. Computational Methods S8-S25
3. NMR Spectra S26-S33
4. HPLC Traces S34-S35

1. Experimental Procedures

1.1 Chemical Synthesis Methods

1.1.1 Friedel-Crafts Alkylation Reaction

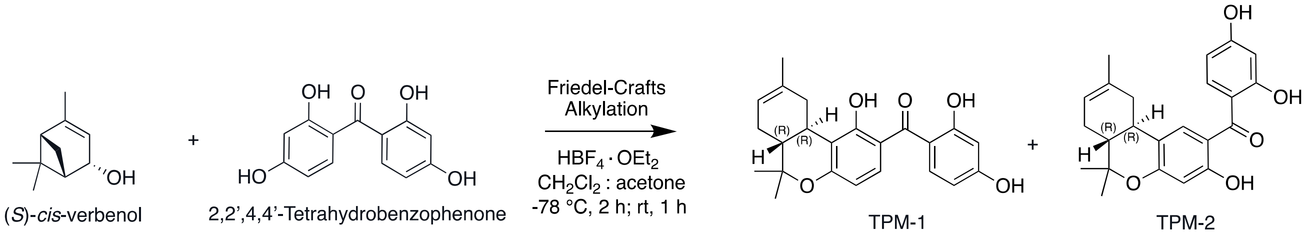

Scheme S1. First step of the BPP synthesis of TPM-1 and TPM-2 via the Friedel-Crafts alkylation chemical reaction.

1.1.2 Terpenoid Aromatization Reaction

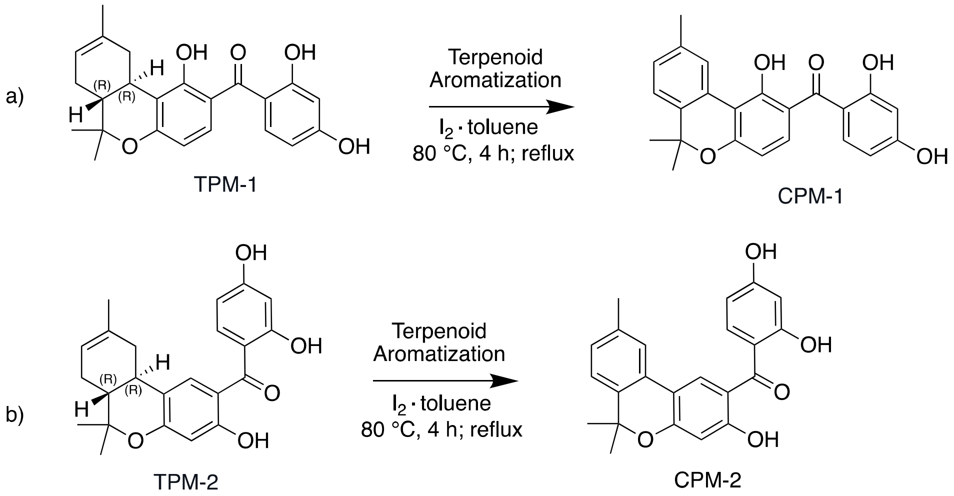

Scheme S2. Second step of the synthesis of a) CPM-1 and b) CPM-2 via the terpenoid aromatization chemical reaction.

1.1.3 Reaction Mechanisms

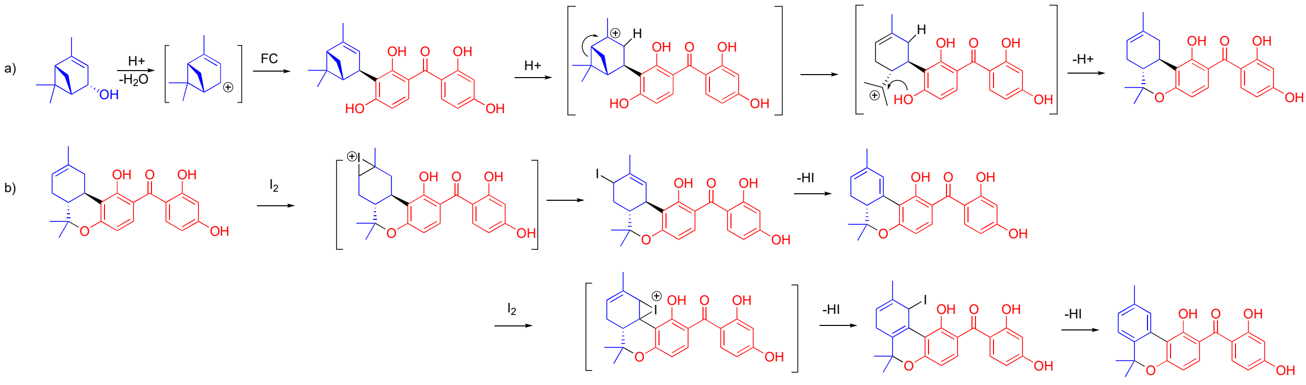

Scheme S3. Plausible reaction mechanisms for the formation of TPMs and CPMs. Shown for TPM-1 and CPM-1.

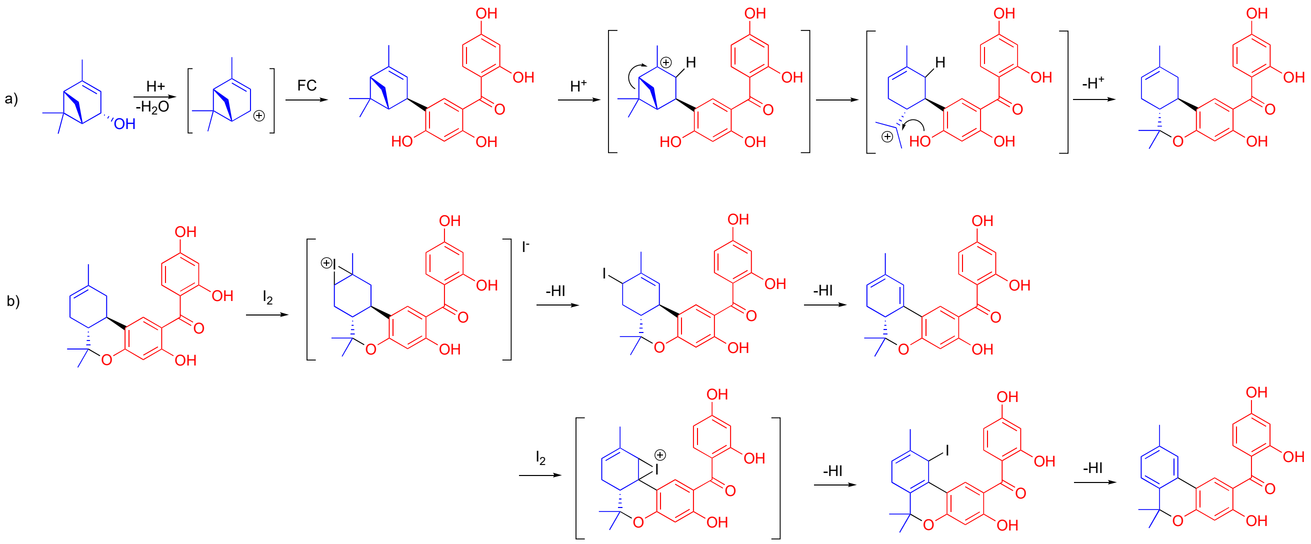

Scheme S4. Plausible reaction mechanisms for the formation of TPMs and CPMs. Shown for TPM-2 and CPM-2.

1.2 General Experimental

1.2.1 Thin Layer Chromatography (TLC)

MiliporeSigma® TLC Silica gel (60 F254) aluminum sheets were utilized to monitor the progression of reactants. Eluted compounds were qualitatively assessed with UV light (λ = 254 nm) or dipping the plates into Vanillin solution (followed by heat gun) or Fast Blue B Salt solution. Hexane/ethyl acetate were used as the mobile phase eluent.

1.2.2 Column Chromatography (CC)

Normal phase column chromatography was conducted using SiliaFlash® P60 (40 – 63 µM, 60 Å) irregular silica gel. Constant mobile phase composition (isocratic chromatography) or gradual increase of the solvent (gradient chromatography) techniques were applied to purify the reaction product. Silica gel behaved as the polar stationary phase, whereas the nonpolar mobile phase was a hexane/ethyl acetate composition.

1.2.3 High Performance Liquid Chromatography (HPLC)

HPLC was performed using a reverse phase column to further purify the product. BPP regioisomers were collected within tight gradient percentages, approximately between 10% H_2O, 90% MeOH to 0% H_2O, 100% MeOH for the mobile phase. The C18 column is the nonpolar stationary phase, whereas the solvent system serves as the polar mobile phase (the opposite of normal phase). Prior to injection into the column, a C18-E SPE sorbent (attached to a 13mm syringe filter with a 0.2 µM PTFE membrane) was used to prepare the sample. All compounds are ≥ 95% pure by HPLC.

Table S1. HPLC conditions used to isolate pure compounds.

**HPLC conditions**	
Instrument	Waters™ 486 Tunable Absorbance Detector and Automated Gradient Controller
Column	Kinetex® LC C18 100 Å Column (250 x 21.2 mm)
Program gradient	Initial gradient on 25% DI H_2O w/ 0.1% formic acid [A] and 75% HPLC MeOH [B]. Going to 0% [A] and 100% [B]
Run time	45 mins
Flow rate	7 mL/min

1.3 *In Vitro* Assays

Antiviral Test

1.3.1 Compound Preparation

Antiviral drug compounds designed and synthesized by researchers in Dr. Mark Hamann's laboratory at the Medical University of South Carolina were sent to Dr. Raymond Schinazi's laboratory at Emory University. *In vitro* antiviral activity against SARS-CoV-2 was evaluated via cytotoxicity, kinetic replication, antiviral evaluation, and antiviral dose response assays. The stock solution of the compounds was prepared in an aliquot of 40 mM using ddH_2O, methanol, or DMSO. Stock solution was diluted in a serial dilution using cell culture medium right before each experiment. Remdesivir was used as an in-house drug control with proven *in vitro* antiviral activity against SARS-CoV-2.

1.3.2 Cell Lines

Vero (African Green Monkey Kidney) and Peripheral Blood Mononuclear (PBM) cell lines were used in this study. Cells were maintained and cultured in MEM containing 10% heat inactivated fetal bovine serum (FBS). The cells were incubated at 37 °C in the presence of 5% CO2. At the time of virus inoculation and antiviral assays, the concentration of FBS was reduced to 2%. SARS-CoV-2 (Isolate USA-WA1/2020) was provided by BEI Resources (Manassas, VA, USA). SARS-CoV-2 has been propagated in each cell line and titrated by $TCID_{50}$ method followed by storage of aliquots at – 80 °C until further use in the experiments.

1.3.3 Virus Kinetic Replication Assays

To determine the kinetic replication of SARS-CoV-2 in each cell line, a confluent monolayer of Vero cells in a 96-well cell culture microplate were inoculated at an MOI of 0.1 and the yield of progeny virus production was assessed in different time points using a specific qRT-PCR for SARS-CoV-2 for each cell line. Briefly, a one-step qRT-PCR was conducted in a final volume of 10 μL containing extracted viral RNA, probe/primer mix (Table 2), and qScript-Tough master mix (Quantibio, Beverly, MA, USA). Quantitative PCR measurement was performed using LightCycler® 480 PCR system (Roche, Manheim, Germany) according to manufacturer's protocol. The protocol for this assay followed the methods utilized by Zandi et al. in "Repurposing Nucleoside Analogs for Human Coronaviruses [1]."

Table S2. Forward and reverse primers isolated from SARS-CoV-2 are listed in the table as their 5′ to 3′ RNA genetic sequence. qRT-PCR was conducted to amplify viral RNA for a kinetic replication assay.

SARS-CoV-2 Primer/Probe	Sequence
Forward Primer	5′-AGA AGA TTG GTT AGA TGA TGA TAG T-3′
Reverse Primer	5′-TTC CAT CTC TAA TTG AGG TTG AAC C-3′
Probe	5′-/56-FAM/TC CTC ACT GCC GTC TTG TTG ACC A/3BHQ_1/-3′

Ames Test

1.3.4 Compound Preparation

CPM-2 was prepared for incubation via 6 series dilution: 10 μM (1/1), 5 μM (1/2), 2.5 μM (1/4), 1.25 (1/8), 0.625 (1/16), and 0.3125 μM (1/32). A 10 mM stock solution of CPM-2 in DMSO was diluted (1/1000) in culture medium to get 10 μM. Diluted concentrations were performed in triplicates in 24-well plates and 1.6 mL of the test substance or the controls were used per well.

1.3.5 Cell Lines

The Ames mutagenicity assays were conducted via *Salmonella* TA98 and TA100 strains. The two strains were cultured in Dr. Zhiwei Ye's laboratory at the Medical University of South Carolina, Department of Cell and Molecular Pharmacology and Experimental Therapeutics, Charleston, South Carolina. TA98 strain was utilized to detect potential frameshift mutations, whereas TA100 strain was utilized to detect potential base-pair substitutions.

Biochemical Target-Validation Test

1.3.6 Expression and Purification of the SARS-CoV-2 2′-*O*-MTase

The SARS-CoV-2 RNA 2′-*O*-MTase is composed of two subunits, Nsp10 and Nsp16. Plasmids encoding the Nsp10 and Nsp16 subunits of the 2′-*O*-MTase were obtained from BEI Resources (NR-52425 and NR-52427) and were used to transform NiCo21(DE3) cells. Expression and purification of Nsp10 and Nsp16 proteins followed the previously described protocol with the following modifications [2]. Cell cultures were induced with 0.5 mM IPTG at an OD 0.8 for Nsp 10 and an OD 1.8 for Nsp 16. Cell growths were harvested and resuspended in lysis buffer (50 mM Tris-HCl pH 8.3, 500 mM NaCl, 10% glycerol, 0.1% IGEPAL) and sonicated. Lysate was clarified through centrifugation and the resulting supernatant was applied to Gold Bio Nickel resin (Gold Biotechnology, Inc., Olivette, MO, USA) and incubated at 4 °C for 2 hours. The nickel resin was washed with 10 mM Tris-HCl pH 8.3, 500 mM NaCl, and 25 mM imidazole, and protein eluted off the resin in 10 mM Tris-HCl pH 8.3, 500 mM NaCl, 1 M imidazole. Nsp 10 was further purified by gel filtration in 10 mM Tris-HCl pH 7.5, 150 mM NaCl, 5 mM $MgCl_2$, 0.5 mM DTT, and 5% glycerol. Proteins were dialyzed into 10 mM Tris-HCl pH 7.5, 150 mM NaCl, 5 mM $MgCl_2$, 0.5 mM DTT, and 5% glycerol. Proteins were concentrated, flash frozen in liquid nitrogen, and stored at −80 °C.

1.3.7 MTaseGlo Kinetic Assay

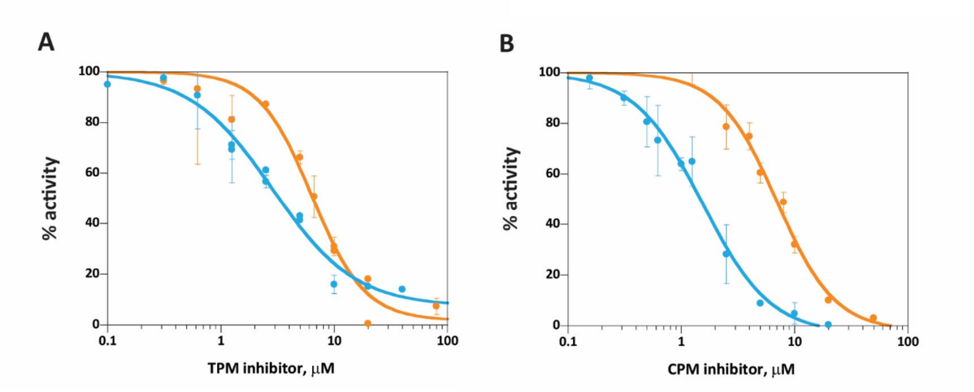

Figure S1. IC_{50} curves for **A)** TPMs 1 (orange) & 2 (blue) , and **B)** CPMs 1 (orange) & 2 (blue), measuring inhibitory activity against the SARS-CoV-2 2′-*O*-MTase.

1.4 *In Silico* Assays

1.4.1 Binding Affinity

The RCSB PDB was utilized to obtain high resolution angstrom (A°) 3D crystal structures for *Coronaviridae* Nsps used for *in silico* computational analyses [3]. The Nsps from SARS-CoV-2 and other coronaviruses derived from X-ray crystallography or NMR spectroscopy from the literature. The data for Nsp10-Nsp16 (6w4h and 3r24) was obtained from "High-resolution structures of the SARS-CoV-2 2′-*O*-methyltransferase reveal strategies for structure-based inhibitor design" and "Biochemical and Structural Insights into the Mechanisms of SARS Coronavirus RNA Ribose 2-O-Methylation by nsp16/nsp10 Protein Complex [2,4]. Drug binding affinities and protein-ligand interaction visuals were obtained via AutoDock Vina [5].

Table S3. Binding affinities (kcal/mol) of synthesized BPPs and major cannabinoids to SARS and MERS coronavirus protein targets: methyltransferase (2′-*O*-MTase, Nsp10-16), 3C-like proteinase protease (3CLpro, Nsp5), RNA-dependent RNA polymerase (RdRp, Nsp12-7-8), and papain-like protease (PLpro, Nsp3). Binding affinity threshold of less than -8.5 kcal/mol is highlighted: Light blue- 2′-O-MTase, Peach- 3CLpro, and Light green- PLpro.

	**SARS-CoV-2**				**SARS-CoV**				**MERS-CoV**		
Protein name	2′-*O*-MTase	3CLpro	RdRp	PLpro	2′-*O*-MTase	3CLpro	RdRp	PLpro	2′-*O*-MTase	3CLpro	PLpro
PDB ID	6w4h	6lu7	7bv2	7cjm	3r24	3v3m	6nur	3e9s	5ynb	4rsp	5w8u
SAM	-7.8	-7.9	-7.1	-9.5	-8.0	-7.5	-7.0	-10.2	-8.0	-7.5	-7.3
Remdesivir	-8.9	-7.4	-7.1	-7.2	-7.8	-8.2	-7.1	-7.3	-8.1	-8.9	-6.9
TPM-1	-9.7	-7.9	-6.7	-7.4	-9.7	-8.3	-6.3	-8.2	-9.8	-9.2	-8.4
CPM-1	-9.8	-8.1	-7.1	-8.2	-9.6	-8.5	-7.4	-8.6	-9.7	-9.3	-8.5
TPM-2	-9.6	-9.5	-7.1	-8.8	-9.0	-8.5	-6.5	-9.3	-9.2	-9.3	-8.8
CPM-2	-9.5	-8.7	-6.8	-8.1	-9.1	-8.9	-6.3	-8.5	-9.4	-9.2	-8.6
CBD	-6.3	-5.8	-5.1	-6.0	-6.8	-6.3	-4.6	-6.5	-6.5	-7.1	-6.6
CBN	-8.1	-7.3	-5.6	-7.1	-8.1	-7.3	-4.9	-8.1	-7.7	-8.2	-6.7
Δ^8-THC	-8.1	-7.2	-5.6	-6.9	-7.9	-7.1	-4.9	-7.9	-7.9	-8.1	-6.7
Δ^9-THC	-8.1	-7.3	-5.4	-8.0	-7.9	-7.1	-5.1	-7.7	-7.9	-8.2	-6.7
PET	-8.9	-7.6	-6.7	-8.0	-9.2	-7.9	-6.8	-9.0	-9.0	-8.2	-8.4

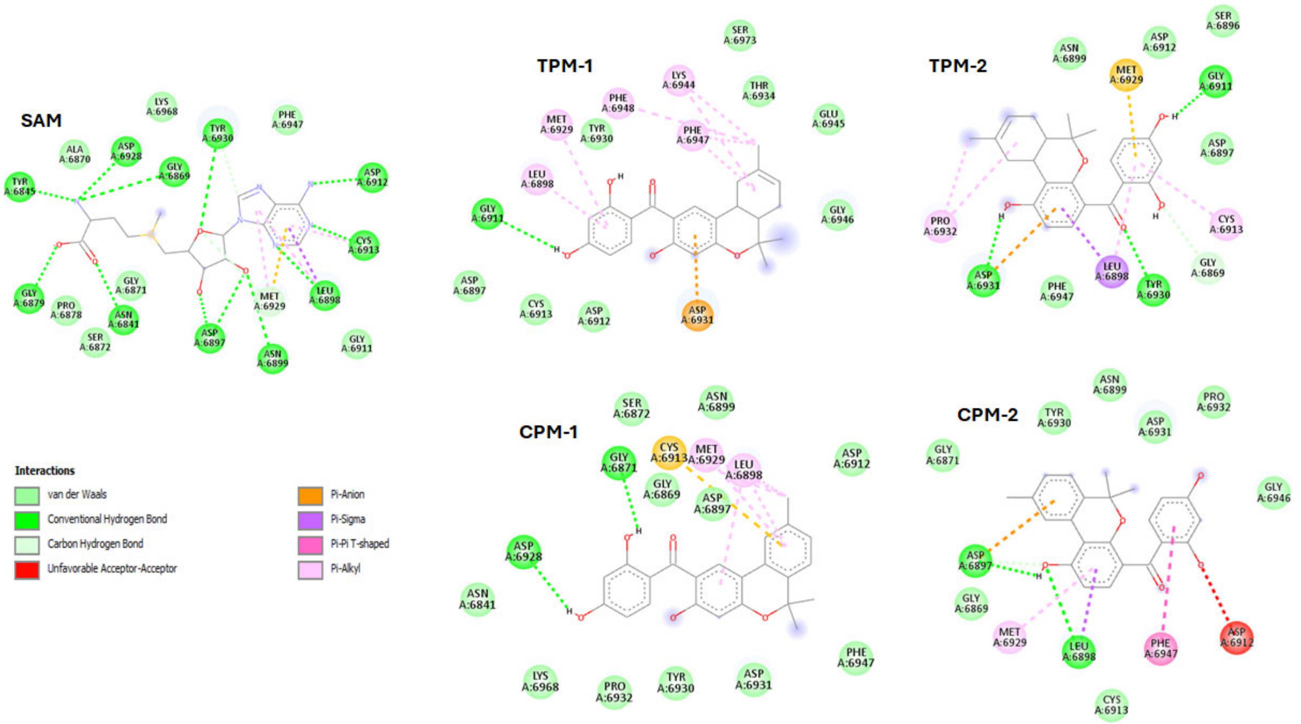

Figure S2. Two-dimensional (2D) *in silico* simulation of the potential inhibition mechanism of SAM, TPM-1, TPM-2, CPM-1, and CPM-2 between the amino acid residues of the 2′-*O*-MTase SARS-CoV-2 protein. Chemical bond interactions are denoted via color.

1.4.2 Molecular Ion Networking (MoIN)

A MoIN analysis was conducted via the Global Natural Products Social (GNPS) molecular networking web-based spectrometry database [6]. The Cytoscape program was utilized to visualize the molecular network and compare the synthesized molecules to some commonly known cannabinoids [7].

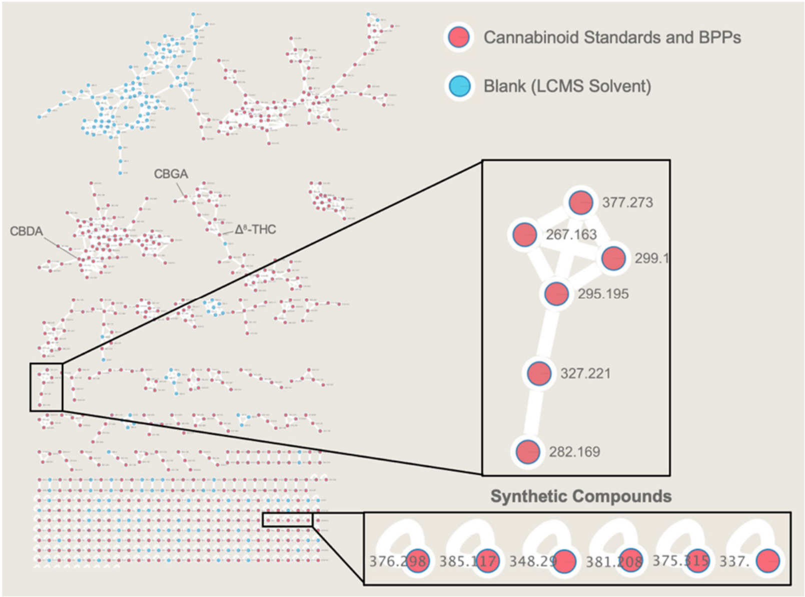

Figure S3. Each cluster of the MoIN is representative of a distinct class of compounds based on fragmentation patterns (molecular "fingerprints") of each molecule's mass peaks.

1.4.3 Software and Databases

Table S4. The computer programs utilized for *in silico* research are listed below.

List of Software/Databases
RCSB PDB
ChemOffice 23
PubChem 2024
AutoDock Vina 1.2.0
AutoDockTools version 1.5.6
Cytoscape 3.9.1
GNPS 1.3.15
Discovery Studio Visualizer v21.1.0.20298
Maestro 2020
LigPrep
DALL·E by OpenAI

2. Computational Methods

2.1 Structures of BPP regioisomers utilized for the DP4+ probability analyses

TPMs

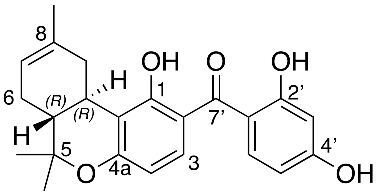

TPM-1

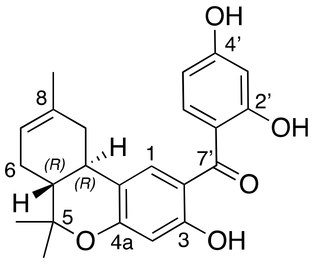

TPM-2

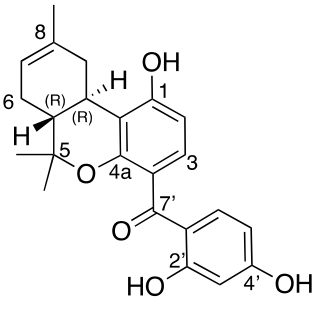

TPM-3

CPMs

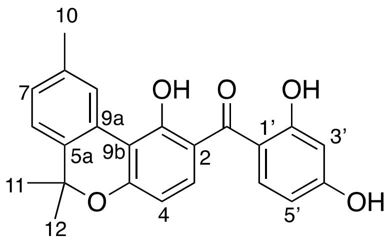

CPM-1

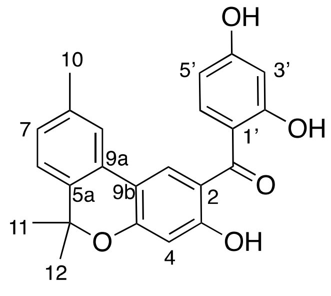

CPM-2

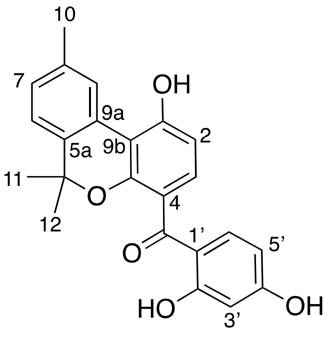

CPM-3

Figure S4. Proposed Structures of potential BPP products: TPM-1, CPM-1, TPM-2, CPM-2, TPM-3 and CPM-3. The stereoisomeric form of the TPMs is of the (*R*,*R*) stereoisomer.

2.2 IUPAC Nomenclature

Nomenclature was assigned following the system guidelines set by the International Union of Pure and Applied Chemistry (IUPAC) for naming and classifying chemical compounds [8].

Table S5. IUPAC nomenclature of novel synthetic TPMs and CPMs, and molecular weight.

**Synthesized TPM / CPM**	**IUPAC (Naming)**	**Molecular Weight (g/mol)**
Tetrahydrophenyl-methanone-1 (TPM-1) $C_{23}H_{24}O_5$	(2,4-dihydroxyphenyl)((6a*R*,10a*R*)-1-hydroxy-6,6,9-trimethyl-6a,7,10,10a-tetrahydro-6*H*-benzo[*c*]chromen-2-yl)methanone	380.440
Chromenephenyl-methanone-1 (CPM-1) $C_{23}H_{20}O_5$	(2,4-dihydroxyphenyl)(1-hydroxy-6,6,9-trimethyl-6*H*-benzo[*c*]chromen-2-yl)methanone	376.408
Tetrahydrophenyl-methanone-2 (TPM-2) $C_{23}H_{24}O_5$	(2,4-dihydroxyphenyl)((6a*R*,10a*R*)-3-hydroxy-6,6,9-trimethyl-6a,7,10,10a-tetrahydro-6*H*-benzo[*c*]chromen-2-yl)methanone	380.440
Chromenephenyl-methanone-1 (CPM-2) $C_{23}H_{20}O_5$	(2,4-dihydroxyphenyl)(3-hydroxy-6,6,9-trimethyl-6*H*-benzo[*c*]chromen-2-yl)methanone	376.408

2.3 NMR Data

2.3.1 Experimental ^{1}H NMR and ^{13}C NMR Chemical Shift Values for TPM-1

Table S6. ^{1}H and ^{13}C NMR assignments (δ) of TPM-1.

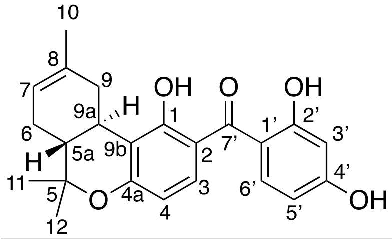

Position	^{1}H NMR in $CDCl_3$[a]	^{13}C NMR in $CDCl_3$[a]
1	12.04 (s, 1H, OH)	161.6
2	-	107.2
3	7.39 (d, *J* = 8.9 Hz, 1H, CH)	132.3
4	6.37 (d, *J* = 8.9 Hz, 1H, CH)	109.4
4a	-	160.6
5	-	78.6
5a	1.91 – 1.77 (m, 3H, CH)	44.6
6	2.20 – 2.14 (m, 1H, CH), 1.91 – 1.77 (m, 1H, CH)	27.8
7	5.46 – 5.43 (m, 1H, CH)	118.9
8	-	134.9
9	3.36 (dd, *J* = 17.1, 4.1 Hz, 1H), 1.91 – 1.77 (m, 1H, CH)	35.5
9a	2.80 (td, *J* = 10.9, 4.8 Hz, 1H, CH)	31.5
9b	-	114.1
10	1.72 (s, 3H, CH_3)	27.4
11	1.43 (s, 3H, CH_3)	23.4
12	1.15 (s, 3H, CH_3)	18.8
1′	-	112.6
2′	5.54 (brs, 1H, OH)	164.1
3′	6.47 (d, *J* = 2.5 Hz, 1H, CH)	104.0
4′	11.13 (s, 1H, OH)	163.9
5′	6.40 (dd, *J* = 8.7, 2.5 Hz, 1H, CH)	114.2
6′	7.53 (d, *J* = 8.7 Hz, 1H, CH)	135.2
7′	-	199.5

[a]Chemical shifts, in ppm, are referenced to the chloroform residual signal (7.26 ppm for ^{1}H and 77.20 ppm for ^{13}C). Coupling constants are reported in hertz (Hz). Splitting patterns are designed as s, singlet; d, doublet; t, triplet; m, multiplet; br, broad. Numbering system follows "Phytocannabinoids: a unified critical inventory" from the Royal Society of Chemistry.

2.3.2 Experimental ^{1}H NMR and ^{13}C NMR Chemical Shift Values for CPM-1

Table S7. ^{1}H and ^{13}C NMR assignments (δ) of CPM-1

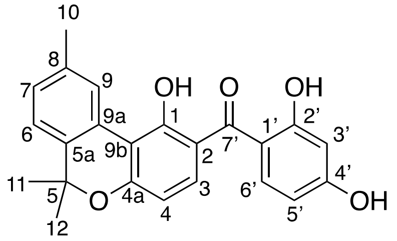

Position	^{1}H NMR in $CDCl_3$[a]	^{13}C NMR in $CDCl_3$[a]
1	12.45 (s, 1H, OH)	161.7
2	-	107.5
3	7.49 (d, *J* = 8.7 Hz, 1H)	128.5
4	6.54 (d, *J* = 8.7 Hz, 1H, CH)	109.5
4a	-	160.2
5	-	78.9
5a	-	135.3
6	7.16 (d, *J* = 7.9 1H, CH)	122.4
7	7.13 (dd, *J* = 7.9, 1.6 Hz, 1H, CH)	126.4
8	-	133.9
9	8.47 (s, 1H, CH_3)	122.4
9a	-	136.0
9b	-	127.5
10	2.41 (s, 3H, CH_3)	21.6
11	1.65 (s, 3H, CH_3)	27.5
12	1.65 (s, 3H, CH_3)	27.5
1′	-	114.1
2′	-	164.4
3′	6.49 (d, *J* = 2.5 Hz, 1H, CH)	104.0
4′	11.13 (s, 1H, OH)	162.4
5′	6.43 (dd, *J* = 8.7, 2.5, 1H, CH)	113.9
6′	7.55 (d, *J* = 8.7 Hz, 1H, CH)	137.2
7′	-	199.95

[a]Chemical shifts, in ppm, are referenced to the chloroform residual signal (7.26 ppm for ^{1}H and 77.20 ppm for ^{13}C). Coupling constants are reported in hertz (Hz). Splitting patterns are designed as s, singlet; d, doublet; t, triplet; m, multiplet; br, broad. Numbering system follows “Phytocannabinoids: a unified critical inventory” from the Royal Society of Chemistry.

2.3.3 Experimental ^{1}H and ^{13}C NMR Chemical Shift Values for TPM-2

Table S8. ^{1}H and ^{13}C NMR assignments (δ) of TPM-2

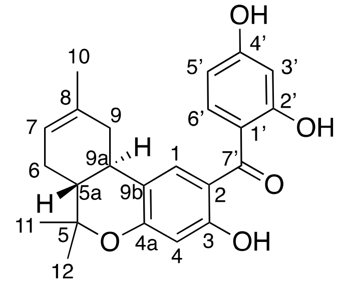

Position	^{1}H NMR in $CDCl_3$[a]	^{13}C NMR in $CDCl_3$[a]
1	7.45 (d, *J* = 1.3 Hz, 1H, CH)	132.2
2	-	107.2
3	11.30 (s, 1H, OH)	161.7
4	6.43 (s, 1H, CH)	104.1
4a	-	160.2
5	-	78.9
5a	2.69 (td, *J* = 11.4, 5.5 Hz, 1H, CH)	42.6
6	2.20 – 2.14 (m, 1H, CH), 1.87 – 1.80 (m, 1H, CH)	27.5
7	5.47 – 5.45 (m, 1H, CH)	117.8
8	-	132.9
9	2.47 (m, 1H, CH), 1.94 – 1.88 (m, 1H, CH)	31.5
9a	2.49 – 2.46 (dd, 1H, CH)	36.6
9b	-	119.9
10	1.71 (s, 3H, CH_3)	27.3
11	1.42 (s, 3H, CH_3)	23.4
12	1.21 (s, 3H, CH_3)	19.7
1′	-	113.7
2′	10.88 (s, 1H, OH)	164.5
3′	6.49 (d, *J* = 2.5 Hz, 2H, CH)	105.2
4′	5.53 (brs, 1H, OH)	162.1
5′	6.43 (dd *J* = 8.7, 2.5 Hz, 1H, CH)	114.2
6′	7.52 (d, *J* = 8.7 Hz, 1H, CH)	135.0
7′	-	199.3

[a]Chemical shifts, in ppm, are referenced to the chloroform residual signal (7.26 ppm for ^{1}H and 77.20 ppm for ^{13}C). Coupling constants are reported in hertz (Hz). Splitting patterns are designed as s, singlet; d, doublet; t, triplet; m, multiplet; br, broad. Numbering system follows "Phytocannabinoids: a unified critical inventory" from the Royal Society of Chemistry.

2.3.4 Experimental ^{1}H and ^{13}C NMR Chemical Shift Values for CPM-2

Table S9. ^{1}H and ^{13}C NMR assignments (δ) of CPM-2

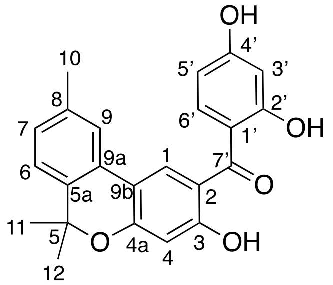

Position	^{1}H NMR in $CDCl_3$[a]	^{13}C NMR in $CDCl_3$[a]
1	7.31 (s, 1H, CH)	135.5
2	-	107.5
3	11.31 (s, 1H, OH)	162.0
4	6.60 (s, 1H, CH)	104.2
4a	-	159.8
5	-	79.3
5a	-	137.7
6	7.14 (d, *J* = 8.7 Hz, 1H, CH)	123.4
7	7.10 (dd, *J* = 8.7 Hz, 1.5 Hz, 1H, CH)	127.6
8	-	135.1
9	7.96 (d, *J* = 1.5 Hz, 1H, CH)	123.4
9a	-	128.5
9b	-	121.9
10	2.37 (s, 3H, CH_3)	21.3
11	1.67 (s, 3H, CH_3)	28.2
12	1.67 (s, 3H, CH_3)	28.2
1′	-	114.1
2′	11.06 (s, 1H, OH)	164.8
3′	6.53 (d, *J* = 2.6 Hz, 1H, CH)	106.3
4′	5.54 (brs, 1H, CH)	163.9
5′	6.47 (dd, *J* = 8.7, 2.6 Hz, 1H, CH)	114.6
6′	7.63 (d, *J* = 8.7 Hz, 1H, CH)	127.2
7′	-	199.4

[a]Chemical shifts, in ppm, are referenced to the chloroform residual signal (7.26 ppm for ^{1}H and 77.20 ppm for ^{13}C). Coupling constants are reported in hertz (Hz). Splitting patterns are designed as s, singlet; d, doublet; t, triplet; m, multiplet; br, broad. Numbering system follows “Phytocannabinoids: a unified critical inventory” from the Royal Society of Chemistry.

2.4 DP4+ Calculations

2.4.1 Mean absolute error (MAE), corrected MAE (CMAE) and DP4+ probability

Table S10. Mean absolute error (MAE), corrected MAE (CMAE) and DP4+ probability analyses (sarotti-nmr.weebly.com) for computed structures TPM-1 (6a*R*,10a*R*), TPM-2 (6a*R*,10a*R*), TPM-3 (6a*R*,10a*R*), TPM-1 (6a*R*,10a*S*), TPM-2 (6a*R*,10a*S*), and TPM-3 (6a*R*,10a*S*) compared with TPM-1 experimental data (Gibbs free energies at the PCM/mPW1PW91/6-311+G(d,p) level were used for the analysis).

	TPM-1 (6a*R*,10a*R*)	TPM-2 (6a*R*,10a*R*)	TPM-3 (6a*R*,10a*R*)	TPM-1 (6a*R*,10a*S*)	TPM-2 (6a*R*,10a*S*)	TPM-3 (6a*R*,10a*S*)	Units
All DP4+ data	100.00	0.00	0.00	0.00	0.00	0.00	%
MAE (carbon)	5.03	6.86	5.13	6.74	6.83	5.17	ppm
CMAE (carbon)	2.15	4.93	3.49	2.68	5.04	3.88	ppm
MAE (proton)	0.17	0.21	0.13	0.49	0.25	0.34	ppm
CMAE (proton)	0.07	0.16	0.11	0.27	0.19	0.30	ppm

Table S11. Mean absolute error (MAE), corrected MAE (CMAE) and DP4+ probability analyses (sarotti-nmr.weebly.com) for computed structures TPM-1 (6a*R*,10a*R*), TPM-2 (6a*R*,10a*R*), TPM-3 (6a*R*,10a*R*), TPM-1 (6a*R*,10a*S*), TPM-2 (6a*R*,10a*S*), and TPM-3 (6a*R*,10a*S*) compared with TPM-2 experimental data (Gibbs free energies at the PCM/mPW1PW91/6-311+G(d,p) level were used for the analysis).

	TPM-1 (6a*R*,10a*R*)	TPM-2 (6a*R*,10a*R*)	TPM-3 (6a*R*,10a*R*)	TPM-1 (6a*R*,10a*S*)	TPM-2 (6a*R*,10a*S*)	TPM-3 (6a*R*,10a*S*)	Units
All DP4+ data	0.00	100.00	0.00	0.00	0.00	0.00	%
MAE (carbon)	7.41	5.20	7.64	8.84	5.07	7.71	ppm
CMAE (carbon)	5.60	2.61	6.21	5.75	2.89	6.35	ppm
MAE (proton)	0.30	0.22	0.27	0.45	0.26	0.32	ppm
CMAE (proton)	0.23	0.17	0.27	0.29	0.19	0.32	ppm

Table S12. Mean absolute error (MAE), corrected MAE (CMAE) and DP4+ probability analyses (sarotti-nmr.weebly.com) for computed structures CPM-1, CPM-2, and CPM-3 compared with CPM-1 experimental data (Gibbs free energies at the PCM/mPW1PW91/6-311+G(d,p) level were used for the analysis).

	CPM-1	CPM-2	CPM-3	Units
All DP4+ data	100.00	0.00	0.00	%
MAE (carbon)	5.84	7.59	5.96	ppm
CMAE (carbon)	3.20	5.91	4.23	ppm
MAE (proton)	0.29	0.35	0.25	ppm
CMAE (proton)	0.12	0.24	0.23	ppm

Table S13 Mean absolute error (MAE), corrected MAE (CMAE) and DP4+ probability analyses (sarotti-nmr.weebly.com) for computed structures CPM-1, CPM-2, and CPM-3 compared with CPM-2 experimental data (Gibbs free energies at the PCM/mPW1PW91/6-311+G(d,p) level were used for the analysis).

	CPM-1	CPM-2	CPM-3	Units

All DP4+ data	0.00	100.00	0.00	%
MAE (carbon)	7.35	5.04	7.88	ppm
CMAE (carbon)	5.35	2.96	5.90	ppm
MAE (proton)	0.34	0.31	0.23	ppm
CMAE (proton)	0.19	0.20	0.20	ppm

2.4.2 DP4+ outcome for isomers compared to experimental data

Table S14. DP4+ outcome for TPM-1 (6a*R*,10a*R*) (Isomer 1), TPM-2 (6a*R*,10a*R*) (Isomer 2), TPM-3 (6a*R*,10a*R*) (Isomer 3), TPM-1 (6a*R*,10a*S*) (Isomer 4), TPM-2 (6a*R*,10a*S*) (Isomer 5), and TPM-3 (6a*R*,10a*S*) (Isomer 6) compared with TPM-1 experimental data. Gibb's free-energy calculations at the PCM/mPW1PW91/6-311+G(d,p) level of theory were used for the analysis.

Functional	Solvent?		Basis Set		Type of Data	
mPW1PW91	PCM		6-311+G(d,p)		Unscaled Shifts	
	Isomer 1	Isomer 2	Isomer 3	Isomer 4	Isomer 5	Isomer 6
sDP4+ (H data)	97.96%	0.03%	2.01%	0.00%	0.00%	0.00%
sDP4+ (C data)	100.00%	0.00%	0.00%	0.00%	0.00%	0.00%
sDP4+ (all data)	100.00%	0.00%	0.00%	0.00%	0.00%	0.00%
uDP4+ (H data)	1.19%	0.05%	98.76%	0.00%	0.00%	0.00%
uDP4+ (C data)	99.98%	0.00%	0.00%	0.02%	0.00%	0.00%
uDP4+ (all data)	100.00%	0.00%	0.00%	0.00%	0.00%	0.00%
DP4+ (H data)	36.89%	0.00%	63.11%	0.00%	0.00%	0.00%
DP4+ (C data)	100.00%	0.00%	0.00%	0.00%	0.00%	0.00%
DP4+ (all data)	100.00%	0.00%	0.00%	0.00%	0.00%	0.00%

Table S15. DP4+ outcome for TPM-1 (6a*R*,10a*R*) (Isomer 1), TPM-2 (6a*R*,10a*R*) (Isomer 2), TPM-3 (6a*R*,10a*R*) (Isomer 3) , TPM-1 (6a*R*,10a*S*) (Isomer 4), TPM-2 (6a*R*,10a*S*) (Isomer 5), and TPM-3 (6a*R*,10a*S*) (Isomer 6) compared with TPM-2 experimental data. Gibb's free-energy calculations at the PCM/mPW1PW91/6-311+G(d,p) level of theory were used for the analysis.

Functional	Solvent?		Basis Set		Type of Data	
mPW1PW91	PCM		6-311+G(d,p)		Unscaled Shifts	
	Isomer 1	Isomer 2	Isomer 3	Isomer 4	Isomer 5	Isomer 6
sDP4+ (H data)	0.04%	99.51%	0.01%	0.00%	0.44%	0.00%
sDP4+ (C data)	0.00%	99.42%	0.00%	0.00%	0.58%	0.00%
sDP4+ (all data)	0.00%	100.00%	0.00%	0.00%	0.00%	0.00%
uDP4+ (H data)	0.11%	95.23%	0.48%	0.00%	4.18%	0.00%
uDP4+ (C data)	0.00%	99.98%	0.00%	0.00%	0.02%	0.00%
uDP4+ (all data)	0.00%	100.00%	0.00%	0.00%	0.00%	0.00%
DP4+ (H data)	0.00%	99.98%	0.00%	0.00%	0.02%	0.00%
DP4+ (C data)	0.00%	100.00%	0.00%	0.00%	0.00%	0.00%
DP4+ (all data)	0.00%	100.00%	0.00%	0.00%	0.00%	0.00%

Table S16. DP4+ outcome for CPM-1 (Isomer 1), CPM-2 (Isomer 2), and CPM-3 (Isomer 3) compared with CPM-1 experimental data. Gibb's free-energy calculations at the PCM/mPW1PW91/6-311+G(d,p) level of theory were used for the analysis.

Functional	Solvent?	Basis Set	Type of Data
mPW1PW91	PCM	6-311+G(d,p)	Unscaled Shifts

	Isomer 1	Isomer 2	Isomer 3	Isomer 4	Isomer 5	Isomer 6
sDP4+ (H data)	99.90%	0.03%	0.07%	-	-	-
sDP4+ (C data)	100.00%	0.00%	0.00%	-	-	-
sDP4+ (all data)	100.00%	0.00%	0.00%	-	-	-
uDP4+ (H data)	1.44%	0.14%	98.42%	-	-	-
uDP4+ (C data)	100.00%	0.00%	0.00%	-	-	-
uDP4+ (all data)	99.94%	0.00%	0.06%	-	-	-
DP4+ (H data)	95.24%	0.00%	4.75%	-	-	-
DP4+ (C data)	100.00%	0.00%	0.00%	-	-	-
DP4+ (all data)	100.00%	0.00%	0.00%	-	-	-

Table S17. DP4+ outcome for CPM-1 (Isomer 1), CPM-2 (Isomer 2), and CPM-3 (Isomer 3) compared with CPM-2 experimental data. Gibb's free-energy calculations at the PCM/mPW1PW91/6-311+G(d,p) level of theory were used for the analysis.

Functional	Solvent?	Basis Set	Type of Data
mPW1PW91	PCM	6-311+G(d,p)	Unscaled Shifts

	Isomer 1	Isomer 2	Isomer 3	Isomer 4	Isomer 5	Isomer 6
sDP4+ (H data)	26.83%	70.74%	2.43%	-	-	-
sDP4+ (C data)	0.00%	100.00%	0.00%	-	-	-
sDP4+ (all data)	0.00%	100.00%	0.00%	-	-	-
uDP4+ (H data)	0.14%	0.56%	99.30%	-	-	-
uDP4+ (C data)	0.00%	100.00%	0.00%	-	-	-
uDP4+ (all data)	0.00%	100.00%	0.00%	-	-	-
DP4+ (H data)	1.33%	13.80%	84.87%	-	-	-
DP4+ (C data)	0.00%	100.00%	0.00%	-	-	-
DP4+ (all data)	0.00%	100.00%	0.00%	-	-	-

2.4.3 Comparison of experimental and calculated NMR chemical shifts of predicted structures

Table S18. Comparison of experimental (Exp) ^{1}H NMR chemical shifts of TPM-2 to calculated structures TPM-1-RR, TPM-2-RR, TPM-3-RR, TPM-1-RS, TPM-2-RS, and TPM-3-RS at the PCM/mPW1PW91/6-311+G(d,p) in $CHCl_3$ and the corrected mean average error (CMAE). The columns labeled, e.g., ∣δTPM-1-RR∣ list the absolute difference for that structure relative to the experimental value. The columns labeled, e.g., Scaled∣δTPM-1-RR∣ list the scaled absolute difference for that structure relative to the experimental value. (All values in ppm.)

position	TPM-1-RR	TPM-2-RR	TPM-3-RR	TPM-1-RS	TPM-2-RS	TPM-3-RS	Exp	∣δTPM-1-RR∣	∣δTPM-2-RR∣	∣δTPM-3-RR∣	∣δTPM-1-RS∣	∣δTPM-2-RS∣	∣δTPM-3-RS∣	Scaled ∣δTPM-1-RR∣	Scaled ∣δTPM-2-RR∣	Scaled ∣δTPM-3-RR∣	Scaled ∣δTPM-1-RS∣	Scaled ∣δTPM-2-RS∣	Scaled ∣δTPM-3-RS∣
1	7.69	7.77	7.37	7.86	7.75	7.40	7.45	0.24	0.32	0.08	0.41	0.30	0.05	0.11	0.00	0.28	0.07	0.01	0.21
4	6.62	6.67	6.47	6.74	6.68	6.49	6.43	0.19	0.24	0.04	0.31	0.25	0.06	0.11	0.01	0.13	0.12	0.00	0.10
6	2.04	1.97	1.95	2.33	1.93	2.23	1.835	0.20	0.13	0.11	0.49	0.10	0.40	0.14	0.18	0.13	0.24	0.03	0.28
6	2.25	2.23	2.18	2.70	2.15	2.58	2.17	0.08	0.06	0.01	0.53	0.02	0.41	0.01	0.09	0.01	0.27	0.09	0.29
7	5.89	5.89	5.89	5.94	5.67	5.80	5.46	0.43	0.43	0.43	0.48	0.21	0.34	0.17	0.23	0.28	0.07	0.00	0.19
9	1.80	1.92	1.87	3.04	2.45	2.94	1.91	0.11	0.01	0.04	1.13	0.54	1.03	0.16	0.06	0.02	0.85	0.46	0.90
9	3.65	2.54	3.56	2.13	2.54	2.12	2.47	1.18	0.07	1.09	0.34	0.07	0.35	1.03	0.08	1.04	0.58	0.02	0.47
10	1.87	1.81	1.87	1.85	1.71	1.82	1.71	0.16	0.10	0.16	0.14	0.00	0.11	0.10	0.16	0.18	0.09	0.06	0.01
11	1.45	1.44	1.15	1.52	1.40	1.17	1.42	0.03	0.02	0.27	0.10	0.02	0.25	0.00	0.10	0.23	0.11	0.06	0.37
12	1.14	1.20	1.05	1.40	1.42	1.22	1.21	0.07	0.01	0.16	0.19	0.21	0.01	0.08	0.08	0.11	0.03	0.17	0.10
9a	2.88	2.73	2.85	3.50	3.35	3.38	2.475	0.41	0.25	0.37	1.03	0.87	0.90	0.30	0.25	0.35	0.73	0.75	0.77
3'	6.52	6.54	6.43	6.66	6.53	6.42	6.49	0.03	0.05	0.06	0.17	0.04	0.07	0.26	0.20	0.23	0.27	0.20	0.22
5'	6.66	6.66	6.53	6.81	6.65	6.50	6.43	0.23	0.23	0.10	0.38	0.22	0.07	0.07	0.02	0.06	0.06	0.02	0.08
6'	7.84	7.80	7.71	8.02	7.75	7.63	7.52	0.32	0.28	0.19	0.50	0.23	0.11	0.04	0.04	0.02	0.01	0.06	0.05
5a	1.81	1.66	1.77	2.09	1.83	1.99	2.69	0.88	1.03	0.92	0.60	0.86	0.70	0.93	0.96	0.90	0.84	0.92	0.82
							**MAE**	**0.30**	**0.22**	**0.27**	**0.45**	**0.26**	**0.32**						
													**CMAE**	**0.23**	**0.17**	**0.27**	**0.29**	**0.19**	**0.32**

Table S19. Comparison of experimental (Exp) ^{13}C NMR chemical shifts of TPM-2 to calculated structures TPM-1-RR, TPM-2-RR, TPM-3-RR, TPM-1-RS, TPM-2-RS, and TPM-3-RS at the PCM/mPW1PW91/6-311+G(d,p) in $CHCl_3$ and the corrected mean average error (CMAE). The columns labeled, e.g., \|δTPM-1-RR\| list the absolute difference for that structure relative to the experimental value. The columns labeled, e.g., Scaled\|δTPM-1-RR\| list the scaled absolute difference for that structure relative to the experimental value. (All values in ppm.)

posi-tion	TPM-1-RR	TPM-2-RR	TPM-3-RR	TPM-1-RS	TPM-2-RS	TPM-3-RS	Exp	|δTPM-1-RR|	|δTPM-2-RR|	|δTPM-3-RR|	|δTPM-1-RS|	|δTPM-2-RS|	|δTPM-3-RS|	Scaled |δTPM-1-RR|	Scaled |δTPM-2-RR|	Scaled |δTPM-3-RR|	Scaled |δTPM-1-RS|	Scaled |δTPM-2-RS|	Scaled |δTPM-3-RS|
1	170.87	139.44	163.98	173.43	138.73	163.08	132.2	38.67	7.24	31.78	41.23	6.53	30.88	30.68	1.46	24.59	30.13	1.19	24.11
2	115.61	116.76	110.00	117.43	116.56	109.23	107.2	8.41	9.56	2.80	10.23	9.36	2.03	3.18	4.56	1.91	3.15	4.95	2.06
3	139.22	169.72	135.28	141.93	170.02	135.13	161.7	22.48	8.02	26.42	19.77	8.32	26.57	28.89	1.19	32.30	28.60	1.66	31.95
4	113.88	109.32	126.96	115.77	109.07	126.56	104.1	9.78	5.22	22.86	11.67	4.97	22.46	4.63	0.48	17.37	4.72	0.87	17.51
5	81.96	82.21	81.27	85.81	81.93	83.12	78.9	3.06	3.31	2.37	6.91	3.03	4.22	0.50	0.50	1.03	2.11	0.08	1.43
6	31.56	31.04	31.52	29.70	26.64	28.96	27.5	4.06	3.54	4.02	2.20	0.86	1.46	3.02	1.50	2.90	1.44	1.49	1.37
7	127.16	128.01	127.55	129.54	128.31	127.12	117.8	9.36	10.21	9.75	11.74	10.51	9.32	3.55	4.82	4.23	3.79	5.61	4.33
8	145.49	143.41	145.32	146.34	140.44	143.09	132.9	12.59	10.51	12.42	13.44	7.54	10.19	5.87	4.59	6.08	4.29	2.12	4.41
9	38.08	39.51	38.66	36.14	34.61	35.84	31.5	6.58	8.01	7.16	4.64	3.11	4.34	5.22	5.68	5.71	3.42	2.15	3.90
10	26.14	25.98	26.20	26.77	26.11	26.27	27.3	1.16	1.32	1.10	0.53	1.19	1.03	1.93	3.18	1.98	1.08	1.80	1.00
11	28.72	28.88	28.30	32.17	27.54	31.18	23.4	5.32	5.48	4.90	8.77	4.14	7.78	4.43	3.52	3.92	7.83	3.48	7.57
12	19.10	19.87	18.70	23.76	27.39	22.85	19.7	0.60	0.17	1.00	4.06	7.69	3.15	1.02	1.48	1.53	3.73	7.03	3.36
1'	116.98	116.93	118.73	119.42	117.08	118.85	113.7	3.28	3.23	5.03	5.72	3.38	5.15	2.02	1.78	0.09	1.50	1.05	0.57
10a	34.60	34.73	35.19	31.39	32.54	31.27	36.6	2.00	1.87	1.41	5.21	4.06	5.33	3.19	4.04	2.70	6.10	4.94	5.54
10b	118.79	122.38	118.05	121.37	118.96	117.92	119.9	1.11	2.48	1.85	1.47	0.94	1.98	6.50	2.71	6.93	5.88	5.45	6.50
2'	171.80	172.02	172.56	175.39	172.06	172.27	164.5	7.30	7.52	8.06	10.89	7.56	7.77	0.73	0.61	0.48	0.34	0.82	0.54
3'	106.25	106.24	105.11	108.48	106.29	105.03	105.2	1.05	1.04	0.09	3.28	1.09	0.17	3.71	3.60	4.58	3.15	2.89	4.05
4'	168.84	168.80	169.27	172.30	168.79	169.15	162.1	6.74	6.70	7.17	10.20	6.69	7.05	1.15	0.09	0.26	0.81	0.08	0.03
4a	167.42	166.95	159.64	169.60	168.03	157.66	160.2	7.22	6.75	0.56	9.40	7.83	2.54	0.60	0.02	7.54	1.42	1.25	9.05
5'	110.62	110.58	110.43	112.92	110.59	110.41	114.2	3.58	3.62	3.77	1.28	3.61	3.79	8.56	8.40	8.51	8.03	7.77	7.94
6'	142.73	142.42	144.46	145.63	142.40	144.72	135	7.73	7.42	9.46	10.63	7.40	9.72	1.14	1.54	3.17	1.53	1.90	3.86
5a	47.06	44.82	47.38	40.52	39.89	39.71	42.6	4.46	2.22	4.78	2.08	2.71	2.89	2.65	0.30	2.93	3.62	3.89	3.52
7'	203.25	203.36	206.28	207.21	203.39	206.87	199.3	3.95	4.06	6.98	7.91	4.09	7.57	5.66	3.92	2.15	5.61	3.98	1.38
							**MAE**	**7.41**	**5.20**	**7.64**	**8.84**	**5.07**	**7.71**						

**CMAE**	**5.60**	**2.61**	**6.21**	**5.75**	**2.89**	**6.35**

Table S20. Comparison of experimental (Exp) ^{1}H NMR chemical shifts of TPM-1 to calculated structures TPM-1-RR, TPM-2-RR, TPM-3-RR, TPM-1-RS, TPM-2-RS, and TPM-3-RS at the PCM/mPW1PW91/6-311+G(d,p) in $CHCl_3$ and the corrected mean average error (CMAE). The columns labeled, e.g., |δTPM-1-RR| list the absolute difference for that structure relative to the experimental value. The columns labeled, e.g., Scaled|δTPM-1-RR| list the scaled absolute difference for that structure relative to the experimental value. (All values in ppm.)

position	TPM-1-RR	TPM-2-RR	TPM-3-RR	TPM-1-RS	TPM-2-RS	TPM-3-RS	Exp	|δTPM-1-RR|	|δTPM-2-RR|	|δTPM-3-RR|	|δTPM-1-RS|	|δTPM-2-RS|	|δTPM-3-RS|	Scaled|δTPM-1-RR|	Scaled|δTPM-2-RR|	Scaled|δTPM-3-RR|	Scaled|δTPM-1-RS|	Scaled|δTPM-2-RS|	Scaled|δTPM-3-RS|
1	6.62	6.67	6.47	6.74	6.68	6.49	6.37	0.25	0.30	0.10	0.37	0.31	0.12	0.01	0.06	0.03	0.07	0.07	0.04
4	7.69	7.77	7.37	7.86	7.75	7.40	7.39	0.30	0.38	0.02	0.47	0.36	0.01	0.01	0.06	0.18	0.03	0.08	0.17
6	2.04	1.97	1.95	2.33	1.93	2.23	1.84	0.20	0.13	0.11	0.49	0.09	0.39	0.12	0.18	0.10	0.25	0.03	0.29
6	2.25	2.23	2.18	2.70	2.15	2.58	2.17	0.08	0.06	0.01	0.53	0.02	0.41	0.01	0.10	0.00	0.28	0.08	0.30
7	5.89	5.89	5.89	5.94	5.67	5.80	5.445	0.45	0.45	0.44	0.49	0.22	0.36	0.21	0.25	0.33	0.08	0.02	0.20
9	1.80	1.92	1.87	3.04	2.45	2.94	1.84	0.04	0.08	0.03	1.20	0.61	1.10	0.10	0.13	0.03	0.93	0.54	0.98
9	3.65	2.54	3.56	2.13	2.54	2.12	3.36	0.29	0.82	0.20	1.23	0.82	1.24	0.14	0.80	0.15	1.45	0.90	1.34
10	1.87	1.81	1.87	1.85	1.71	1.82	1.72	0.15	0.09	0.15	0.13	0.01	0.10	0.08	0.16	0.15	0.08	0.06	0.00
11	1.45	1.44	1.15	1.52	1.40	1.17	1.43	0.02	0.01	0.28	0.09	0.03	0.26	0.03	0.09	0.27	0.10	0.07	0.36
12	1.14	1.20	1.05	1.40	1.42	1.22	1.15	0.01	0.05	0.10	0.25	0.27	0.07	0.04	0.15	0.08	0.06	0.23	0.02
9a	2.88	2.73	2.85	3.50	3.35	3.38	2.8	0.08	0.07	0.05	0.70	0.55	0.58	0.03	0.07	0.02	0.41	0.43	0.46
3'	6.52	6.54	6.43	6.66	6.53	6.42	6.47	0.05	0.07	0.04	0.19	0.06	0.05	0.21	0.17	0.17	0.26	0.17	0.21
5'	6.66	6.66	6.53	6.81	6.65	6.50	6.4	0.26	0.26	0.13	0.41	0.25	0.10	0.00	0.02	0.00	0.04	0.02	0.06
6'	7.84	7.80	7.71	8.02	7.75	7.63	7.53	0.31	0.27	0.18	0.49	0.22	0.10	0.01	0.05	0.01	0.02	0.06	0.07
5a	1.81	1.66	1.77	2.09	1.83	1.99	1.84	0.03	0.18	0.07	0.25	0.01	0.15	0.10	0.11	0.06	0.03	0.06	0.05
							**MAE**	**0.17**	**0.21**	**0.13**	**0.49**	**0.25**	**0.34**						
													**CMAE**	**0.07**	**0.16**	**0.11**	**0.27**	**0.19**	**0.30**

Table S21. Comparison of experimental (Exp) ^{13}C NMR chemical shifts of TPM-1 to calculated structures TPM-1-RR, TPM-2-RR, TPM-3-RR, TPM-1-RS, TPM-2-RS, and TPM-3-RS at the PCM/mPW1PW91/6-311+G(d,p) in $CHCl_3$ and the corrected mean average error (CMAE). The columns labeled, e.g., |δTPM-1-RR| list the absolute difference for that structure relative to the experimental value. The columns labeled, e.g., Scaled|δTPM-1-RR| list the scaled absolute difference for that structure relative to the experimental value. (All values in ppm.)

position	TPM-1-RR	TPM-2-RR	TPM-3-RR	TPM-1-RS	TPM-2-RS	TPM-3-RS	Exp	|δTPM-1-RR|	|δTPM-2-RR|	|δTPM-3-RR|	|δTPM-1-RS|	|δTPM-2-RS|	|δTPM-3-RS|	Scaled |δTPM-1-RR|	Scaled |δTPM-2-RR|	Scaled |δTPM-3-RR|	Scaled |δTPM-1-RS|	Scaled |δTPM-2-RS|	Scaled |δTPM-3-RS|
1	170.87	139.44	163.98	173.43	138.73	163.08	161.6	9.27	22.16	2.38	11.83	22.87	1.48	2.48	28.17	3.76	1.86	28.46	4.30
2	115.61	116.76	110.00	117.43	116.56	109.23	112.6	3.01	4.16	2.60	4.83	3.96	3.37	1.93	0.84	7.12	1.97	0.46	7.28
3	139.22	169.72	135.28	141.93	170.02	135.13	132.3	6.92	37.42	2.98	9.63	37.72	2.83	1.18	30.05	2.30	1.45	30.48	1.98
4	113.88	109.32	126.96	115.77	109.07	126.56	109.4	4.48	0.08	17.56	6.37	0.33	17.16	0.41	4.74	12.53	0.33	4.36	12.65
5	81.96	82.21	81.27	85.81	81.93	83.12	78.6	3.36	3.61	2.67	7.21	3.33	4.52	0.47	0.16	1.00	2.20	0.73	1.52
6	31.56	31.04	31.52	29.70	26.64	28.96	27.8	3.76	3.24	3.72	1.90	1.16	1.16	1.62	2.07	1.54	0.06	0.84	0.04
7	127.16	128.01	127.55	129.54	128.31	127.12	118.9	8.26	9.11	8.65	10.64	9.41	8.22	2.92	3.61	3.60	3.15	4.37	3.69
8	145.49	143.41	145.32	146.34	140.44	143.09	134.9	10.59	8.51	10.42	11.44	5.54	8.19	4.64	2.32	4.84	3.01	0.15	3.10
9	38.08	39.51	38.66	36.14	34.61	35.84	35.5	2.58	4.01	3.16	0.64	0.89	0.34	0.22	2.47	0.77	1.56	0.99	1.02
10	26.14	25.98	26.20	26.77	26.11	26.27	27.4	1.26	1.42	1.20	0.63	1.29	1.13	3.22	2.36	3.22	2.30	0.94	2.17
11	28.72	28.88	28.30	32.17	27.54	31.18	23.4	5.32	5.48	4.90	8.77	4.14	7.78	3.28	4.41	2.82	6.79	4.42	6.58
12	19.10	19.87	18.70	23.76	27.39	22.85	18.8	0.30	1.07	0.10	4.96	8.59	4.05	1.43	0.41	1.89	3.45	8.87	3.13
1'	116.98	116.93	118.73	119.42	117.08	118.85	107.2	9.78	9.73	11.53	12.22	9.88	11.65	4.78	4.72	6.74	5.31	5.43	7.40
9a	34.60	34.73	35.19	31.39	32.54	31.27	31.5	3.10	3.23	3.69	0.11	1.04	0.23	0.85	1.90	1.40	2.05	1.05	1.44
9b	118.79	122.38	118.05	121.37	118.96	117.92	114.1	4.69	8.28	3.95	7.27	4.86	3.82	0.37	3.03	0.81	0.25	0.31	0.39
2'	171.80	172.02	172.56	175.39	172.06	172.27	164.1	7.70	7.92	8.46	11.29	7.96	8.17	0.88	0.45	2.07	1.22	0.61	2.07
3'	106.25	106.24	105.11	108.48	106.29	105.03	104	2.25	2.24	1.11	4.48	2.29	1.03	2.38	2.29	3.27	1.81	1.59	2.73
4'	168.84	168.80	169.27	172.30	168.79	169.15	163.9	4.94	4.90	5.37	8.40	4.89	5.25	1.79	2.42	0.92	1.50	2.29	0.74
4a	167.42	166.95	159.64	169.60	168.03	157.66	160.6	6.82	6.35	0.96	9.00	7.43	2.94	0.14	0.89	6.96	0.75	0.29	8.54
5'	110.62	110.58	110.43	112.92	110.59	110.41	114.2	3.58	3.62	3.77	1.28	3.61	3.79	8.36	8.34	8.31	7.83	7.72	7.74
6'	142.73	142.42	144.46	145.63	142.40	144.72	135.2	7.53	7.22	9.26	10.43	7.20	9.52	1.67	1.08	3.71	2.04	1.41	4.38
5a	47.06	44.82	47.38	40.52	39.89	39.71	44.6	2.46	0.22	2.78	4.08	4.71	4.89	0.20	1.57	0.13	6.53	5.09	6.39
7'	203.25	203.36	206.28	207.21	203.39	206.87	199.5	3.75	3.86	6.78	7.71	3.89	7.37	4.13	5.01	0.63	4.16	5.12	0.07
							**MAE**	**5.03**	**6.86**	**5.13**	**6.74**	**6.83**	**5.17**						

**CMAE**	**2.15**	**4.93**	**3.49**	**2.68**	**5.04**	**3.88**

Table S22. Comparison of experimental (Exp) ^{1}H NMR chemical shifts of CPM-2 to calculated structures CPM-1, CPM-2, and CPM-3 at the PCM/mPW1PW91/6-311+G(d,p) in $CHCl_3$ and the corrected mean average error (CMAE). The columns labeled, e.g., |δTPM-1-RR| list the absolute difference for that structure relative to the experimental value. The columns labeled, e.g., Scaled|δTPM-1-RR| list the scaled absolute difference for that structure relative to the experimental value. (All values in ppm.)

Position	CPM-1	CPM-2	CPM-3	Exp	|δCPM-1|	|δCPM-2|	|δCPM-3|	Scaled|δCPM-1|	Scaled|δCPM-2|	Scaled|δCPM-3|
1	7.77	8.33	7.59	7.31	0.46	1.02	0.28	0.01	0.63	0.04
4	6.78	6.83	7.03	6.6	0.18	0.23	0.43	0.19	0.06	0.22
6	7.56	7.53	7.65	7.14	0.42	0.39	0.51	0.01	0.06	0.26
7	7.56	7.51	7.62	7.1	0.46	0.41	0.52	0.03	0.08	0.27
9	9.08	7.75	8.02	7.96	1.12	0.21	0.06	0.57	0.56	0.22
10	2.53	2.45	2.55	2.37	0.16	0.08	0.18	0.15	0.07	0.26
11	1.42	1.90	1.61	1.67	0.25	0.23	0.06	0.16	0.25	0.08
12	1.91	1.43	1.29	1.67	0.24	0.24	0.38	0.29	0.19	0.23
3'	6.55	6.58	6.53	6.53	0.02	0.05	0.00	0.32	0.22	0.18
5'	6.69	6.74	6.37	6.47	0.22	0.27	0.10	0.14	0.01	0.27
6'	7.86	7.94	7.66	7.63	0.23	0.31	0.03	0.22	0.05	0.22
				**MAE**	**0.34**	**0.31**	**0.23**			
							**CMAE**	**0.19**	**0.20**	**0.20**

Table S23. Comparison of experimental (Exp) ^{13}C NMR chemical shifts of CPM-2 to calculated structures CPM-1, CPM-2, and CPM-3 at the PCM/mPW1PW91/6-311+G(d,p) in $CHCl_3$ and the corrected mean average error (CMAE). The columns labeled, e.g., |δTPM-1-RR| list the absolute difference for that structure relative to the experimental value. The columns labeled, e.g., Scaled|δTPM-1-RR| list the scaled absolute difference for that structure relative to the experimental value. (All values in ppm.)

Position	CPM-1	CPM-2	CPM-3	Exp	|δCPM-1-δExp|	|δCPM-2-δExp|	|δCPM-3-δExp|	Scaled|δCPM-1-δExp|	Scaled|δCPM-2-δExp|	Scaled|δCPM-3-δExp|
1	168.89	134.41	162.18	135.46	33.43	1.05	26.72	25.96	5.91	19.91
2	116.96	117.55	114.15	107.5	9.46	10.05	6.65	5.15	5.88	2.67
3	140.84	171.61	137.70	162	21.16	9.61	24.30	26.92	3.20	29.66
4	113.92	110.52	126.92	104.2	9.72	6.32	22.72	5.59	2.45	17.99
5	82.75	83.29	82.65	79.3	3.45	3.99	3.35	1.22	1.25	1.23
6	128.08	129.08	130.46	123.4	4.68	5.68	7.06	0.31	1.04	2.12
7	134.06	133.86	134.54	127.62	6.44	6.24	6.92	1.09	1.40	1.74
8	144.27	145.06	146.12	135.12	9.15	9.94	11.00	3.18	4.64	5.13
9	132.93	127.19	127.13	123.4	9.53	3.79	3.73	4.25	0.78	1.01
10	23.37	23.23	23.63	21.3	2.07	1.93	2.33	3.45	1.68	3.69
11	28.16	27.93	27.43	28.2	0.04	0.27	0.77	1.05	0.72	0.37
12	27.96	29.50	26.67	28.2	0.24	1.30	1.53	0.86	0.79	0.34
1'	116.93	116.84	118.80	114.1	2.83	2.74	4.70	1.48	1.39	0.45
10a	132.46	133.13	132.60	128.5	3.96	4.63	4.10	1.29	0.18	0.96
10b	116.06	119.04	115.30	121.91	5.85	2.87	6.61	10.11	7.09	10.65
2'	172.07	172.20	172.72	164.8	7.27	7.40	7.92	0.38	0.97	0.49
3'	106.38	106.36	105.56	106.3	0.08	0.06	0.74	3.59	3.64	4.21
4'	169.14	169.13	169.50	163.94	5.20	5.19	5.56	2.28	1.11	1.68
4a	167.09	166.64	158.74	159.82	7.27	6.82	1.08	0.08	0.62	7.69
5'	110.92	110.98	110.26	114.6	3.68	3.62	4.34	7.62	7.51	8.08
6'	142.86	142.45	144.23	127.17	15.69	15.28	17.06	9.80	10.09	11.31
5a	141.20	140.68	144.15	137.71	3.49	2.97	6.44	2.29	2.16	0.69
7'	203.85	203.53	205.14	199.42	4.43	4.11	5.72	5.16	3.62	3.62
				**MAE**	**7.35**	**5.04**	**7.88**			
							**CMAE**	**5.35**	**2.96**	**5.90**

Table S24. Comparison of experimental (Exp) ^{1}H NMR chemical shifts of CPM-1 to calculated structures CPM-1, CPM-2, and CPM-3 at the PCM/mPW1PW91/6-311+G(d,p) in $CHCl_3$ and the corrected mean average error (CMAE). The columns labeled, e.g., |δTPM-1-RR| list the absolute difference for that structure relative to the experimental value. The columns labeled, e.g., Scaled|δTPM-1-RR| list the scaled absolute difference for that structure relative to the experimental value. (All values in ppm.)

Position	CPM-1	CPM-2	CPM-3	Exp	|δCPM-1|	|δCPM-2|	|δCPM-3|	Scaled|δCPM-1|	Scaled|δCPM-2|	Scaled|δCPM-3|
3	7.77	8.33	7.59	7.49	0.28	0.84	0.10	0.08	0.54	0.06
2	6.78	6.83	7.03	6.54	0.24	0.29	0.49	0.06	0.07	0.35
6	7.56	7.53	7.65	7.16	0.40	0.37	0.49	0.05	0.11	0.32
7	7.56	7.51	7.62	7.13	0.43	0.38	0.49	0.09	0.12	0.33
9	9.08	7.75	8.02	8.47	0.61	0.72	0.45	0.18	0.99	0.64
10	2.53	2.45	2.55	2.41	0.12	0.04	0.14	0.08	0.02	0.20
11	1.42	1.90	1.61	1.65	0.23	0.25	0.04	0.21	0.25	0.06
12	1.91	1.43	1.29	1.65	0.26	0.22	0.36	0.26	0.20	0.25
3'	6.55	6.58	6.53	6.49	0.06	0.09	0.04	0.22	0.12	0.08
5'	6.69	6.74	6.37	6.43	0.26	0.31	0.06	0.03	0.09	0.17
6'	7.86	7.94	7.66	7.55	0.31	0.39	0.11	0.05	0.11	0.06
				**MAE**	**0.29**	**0.35**	**0.25**			
							**CMAE**	**0.12**	**0.24**	**0.23**

Table S25. Comparison of experimental (Exp) ^{13}C NMR chemical shifts of CPM-1 to calculated structures CPM-1, CPM-2, and CPM-3 at the PCM/mPW1PW91/6-311+G(d,p) in $CHCl_3$ and the corrected mean average error (CMAE). The columns labeled, e.g., |δTPM-1-RR| list the absolute difference for that structure relative to the experimental value. The columns labeled, e.g., Scaled|δTPM-1-RR| list the scaled absolute difference for that structure relative to the experimental value. (All values in ppm.)

Position	CPM-1	CPM-2	CPM-3	Exp	|δCPM-1-δExp|	|δCPM-2-δExp|	|δCPM-3 δExp|	Scaled|δCPM-1-δExp|	Scaled|δCPM-2-δExp|	Scaled|δCPM-3-δExp|
1	168.89	134.41	162.18	161.7	7.19	27.29	0.48	0.92	32.05	5.23
2	116.96	117.55	114.15	107.5	9.46	10.05	6.65	5.43	6.38	2.91
3	140.84	171.61	137.70	128.5	12.34	43.11	9.20	7.28	35.93	4.50
4	113.92	110.52	126.92	109.5	4.42	1.02	17.42	0.52	2.19	13.15
5	82.75	83.29	82.65	78.9	3.85	4.39	3.75	1.30	2.95	1.31
6	128.08	129.08	130.46	122.4	5.68	6.68	8.06	1.17	2.26	3.65
7	134.06	133.86	134.54	126.4	7.66	7.46	8.14	2.90	2.73	3.56
8	144.27	145.06	146.12	133.9	10.37	11.16	12.22	5.16	5.70	7.17
9	132.93	127.19	127.13	122.4	10.53	4.79	4.73	5.81	0.49	0.46
10	23.37	23.23	23.63	21.6	1.77	1.63	2.03	1.78	4.09	2.01
11	28.16	27.93	27.43	27.5	0.66	0.43	0.07	0.47	2.58	0.24
12	27.96	29.50	26.67	27.5	0.46	2.00	0.83	0.28	4.05	0.97
1'	116.93	116.84	118.80	114.1	2.83	2.74	4.70	1.20	0.88	0.77
10a	132.46	133.13	132.60	136	3.54	2.87	3.40	8.24	7.55	7.89
10b	116.06	119.04	115.30	127.5	11.44	8.46	12.20	15.43	12.22	15.98
2'	172.07	172.20	172.72	164.4	7.67	7.80	8.32	1.27	0.57	2.18
3'	106.38	106.36	105.56	104	2.38	2.36	1.56	1.19	0.59	1.83
4'	169.14	169.13	169.50	162.4	6.74	6.73	7.10	0.46	0.29	1.09
4a	167.09	166.64	158.74	160.2	6.89	6.44	1.46	0.70	0.42	7.03
5'	110.92	110.98	110.26	113.9	2.98	2.92	3.64	6.75	6.16	7.22
6'	142.86	142.45	144.23	137.2	5.66	5.25	7.03	0.51	0.03	2.06
5a	141.20	140.68	144.15	135.3	5.90	5.38	8.85	0.83	0.20	3.88
7'	203.85	203.53	205.14	199.95	3.90	3.58	5.19	3.88	5.68	2.28
				**MAE**	**5.84**	**7.59**	**5.96**			
							**CMAE**	**3.20**	**5.91**	**4.23**

3. NMR Spectra

3.1 Annotated ^{1}H and ^{13}C NMR Spectra for TPM-1

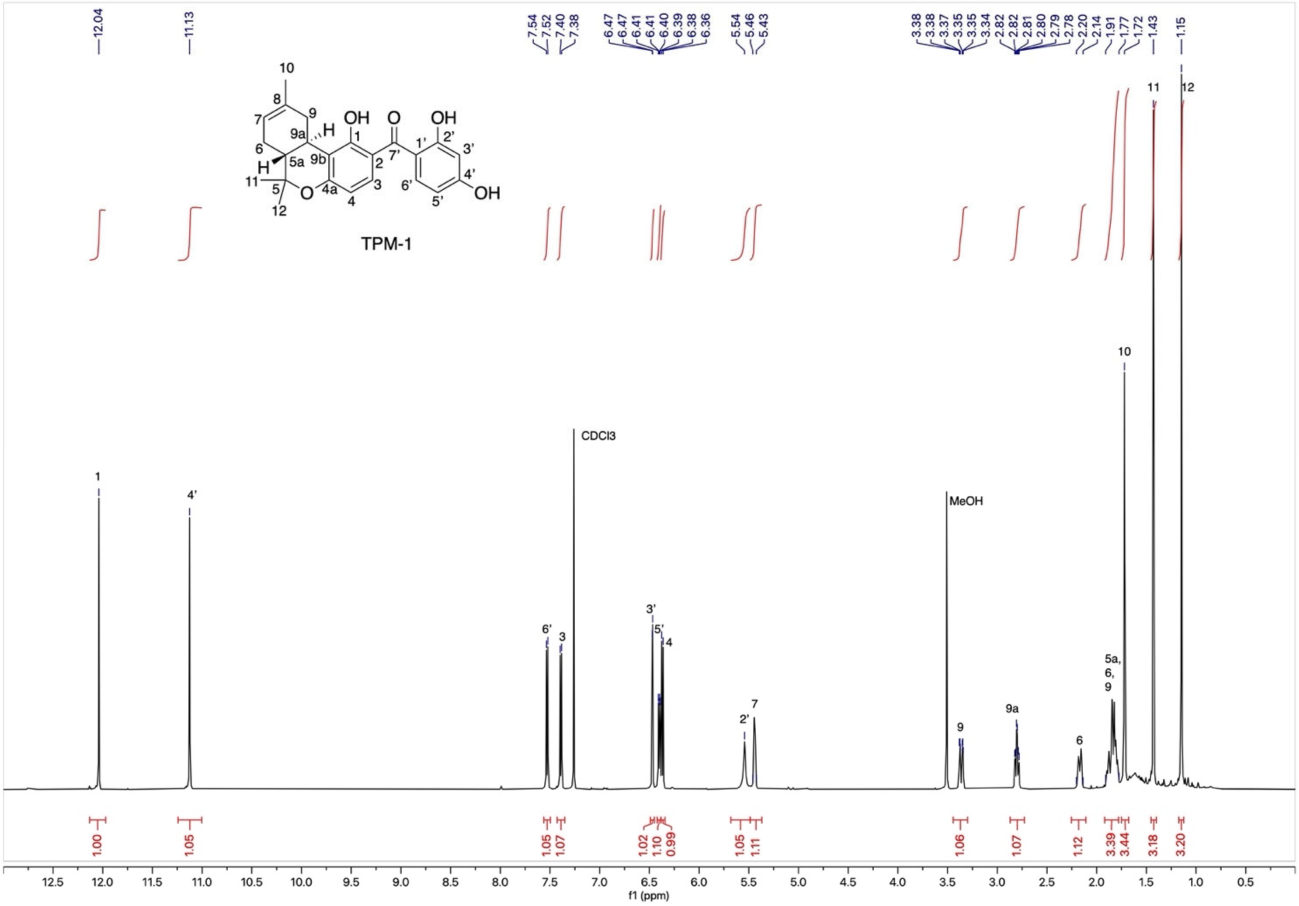

Figure S5. ^{1}H NMR ($CDCl_3$, 600 MHz) of TPM-1

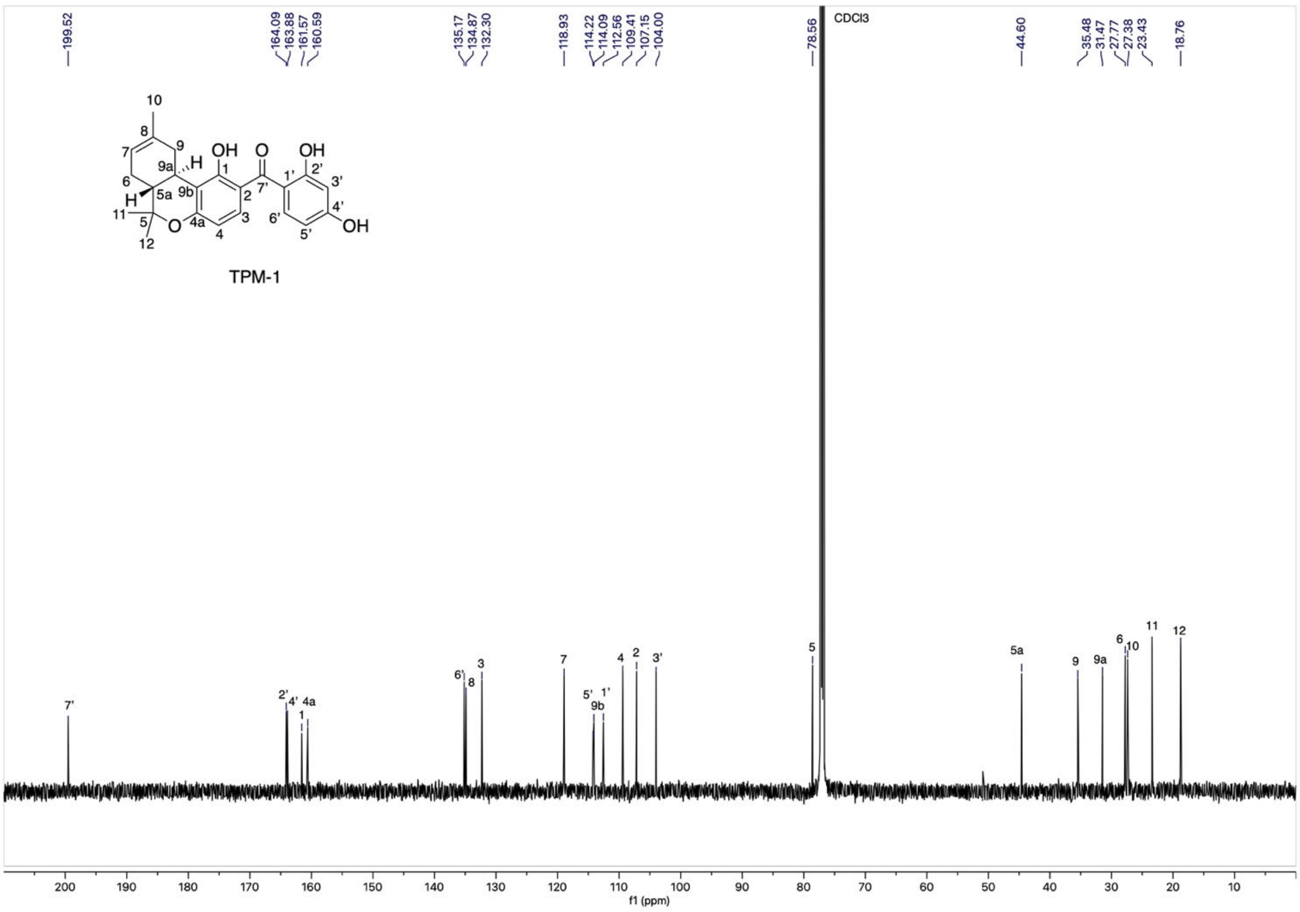

Figure S6. ^{13}C NMR ($CDCl_3$, 150 MHz) of TPM-1

3.2 Annotated ^{1}H and ^{13}C NMR Spectra for CPM-1

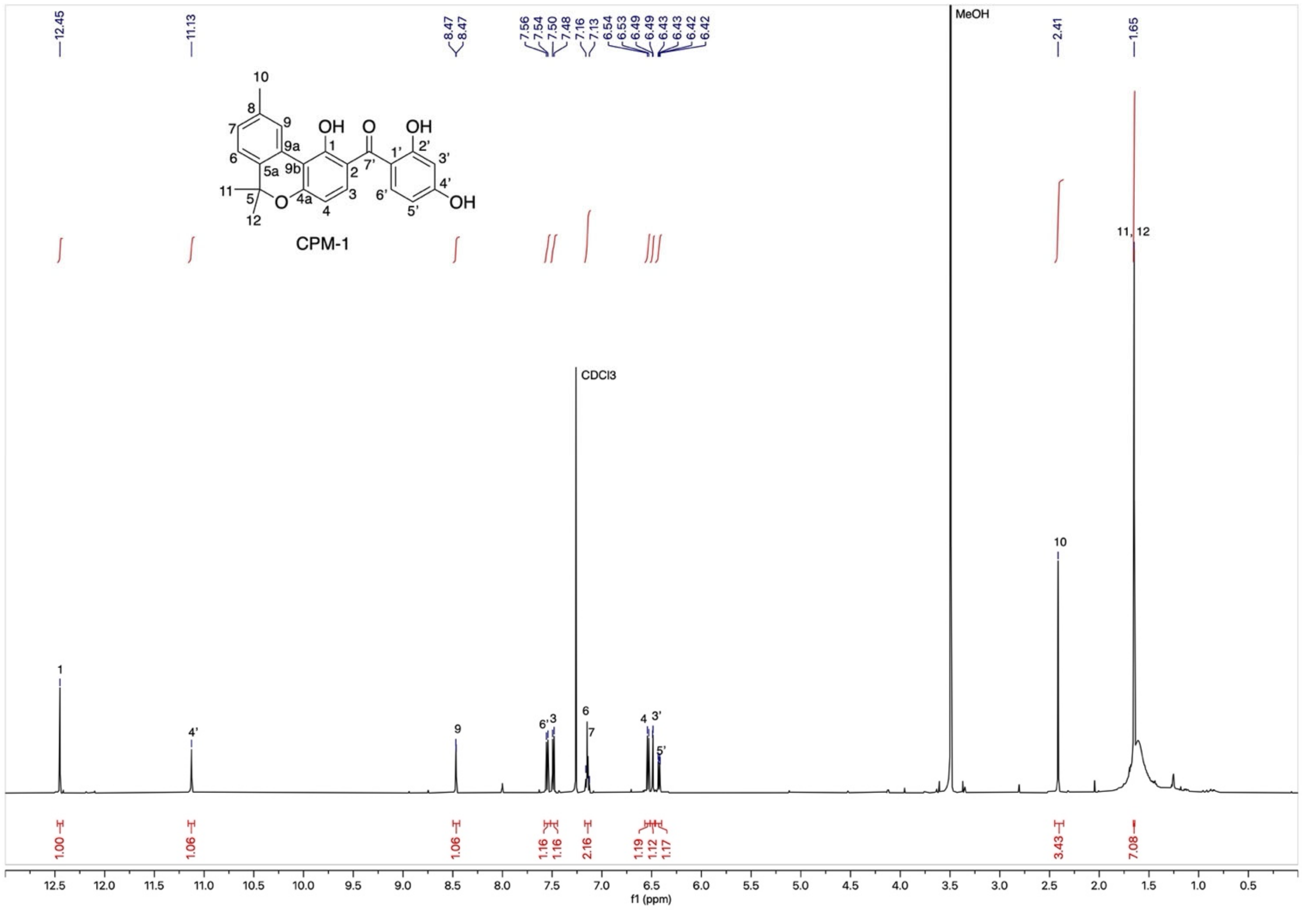

Figure S7. ^{1}H NMR ($CDCl_3$, 600 MHz) of CPM-1

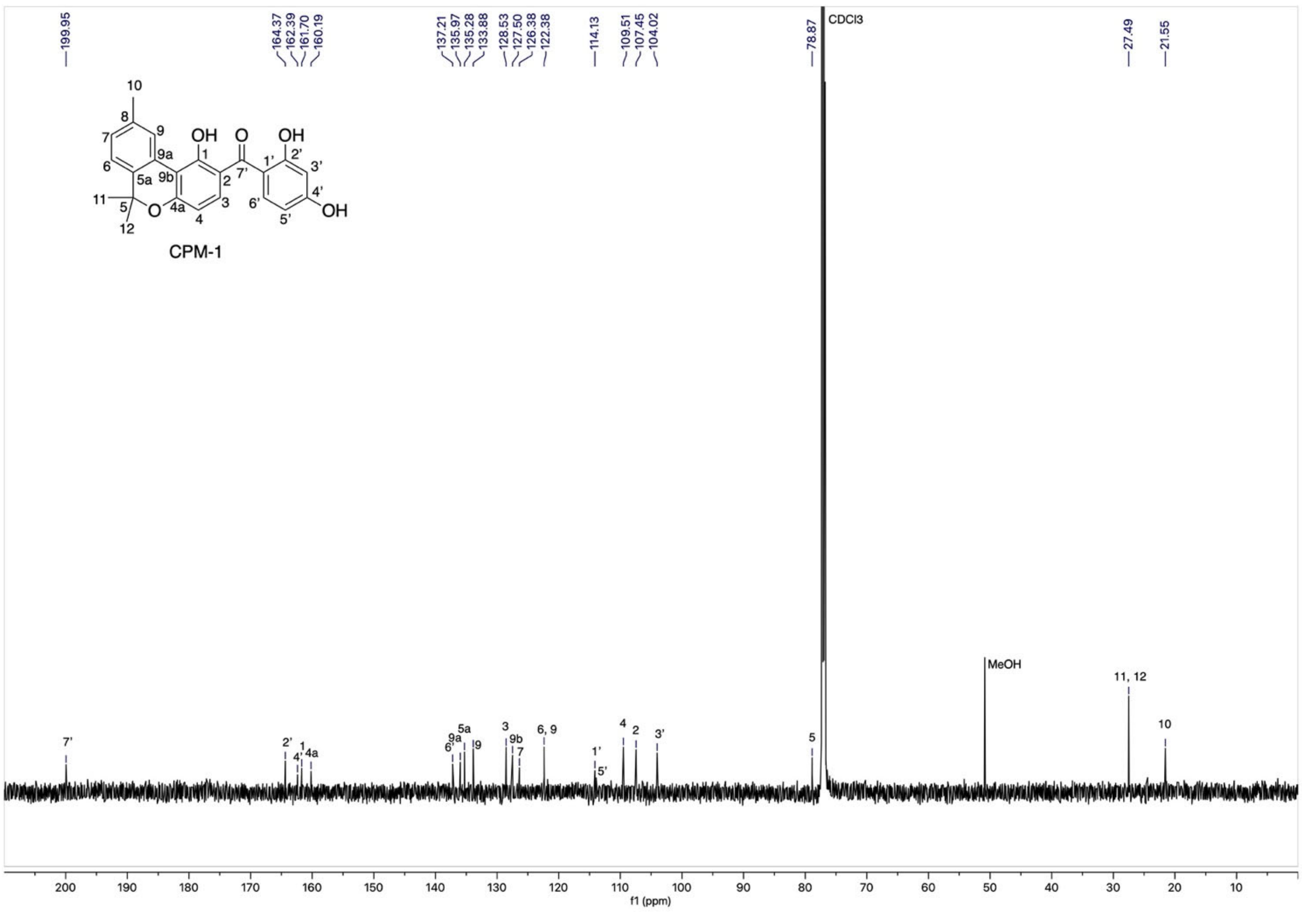

Figure S8. ^{13}C NMR ($CDCl_3$, 150 MHz) of CPM-1

3.3 Annotated ^{1}H and ^{13}C NMR Spectra for TPM-2

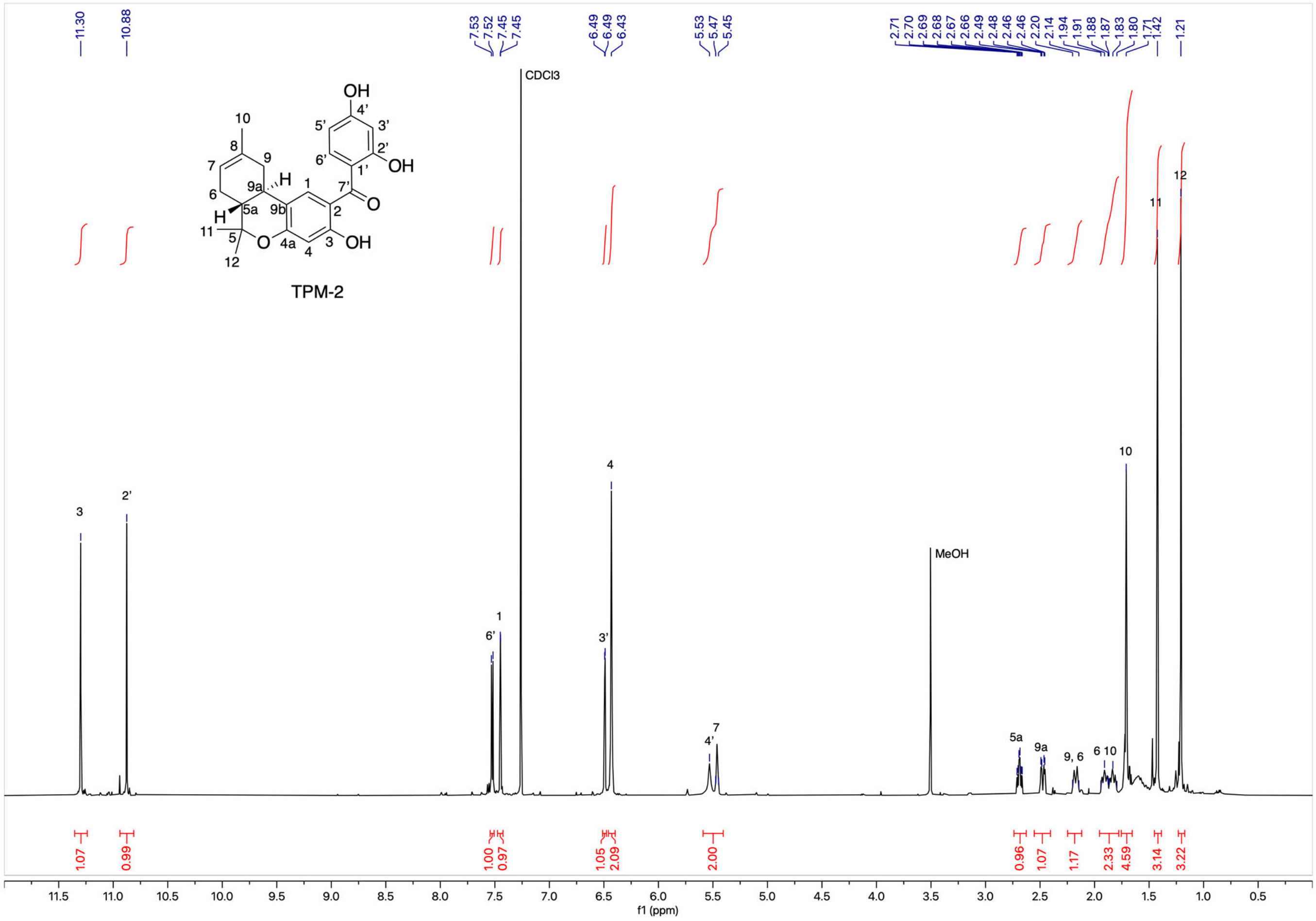

Figure S9. ^{1}H NMR ($CDCl_3$, 600 MHz) of TPM-2

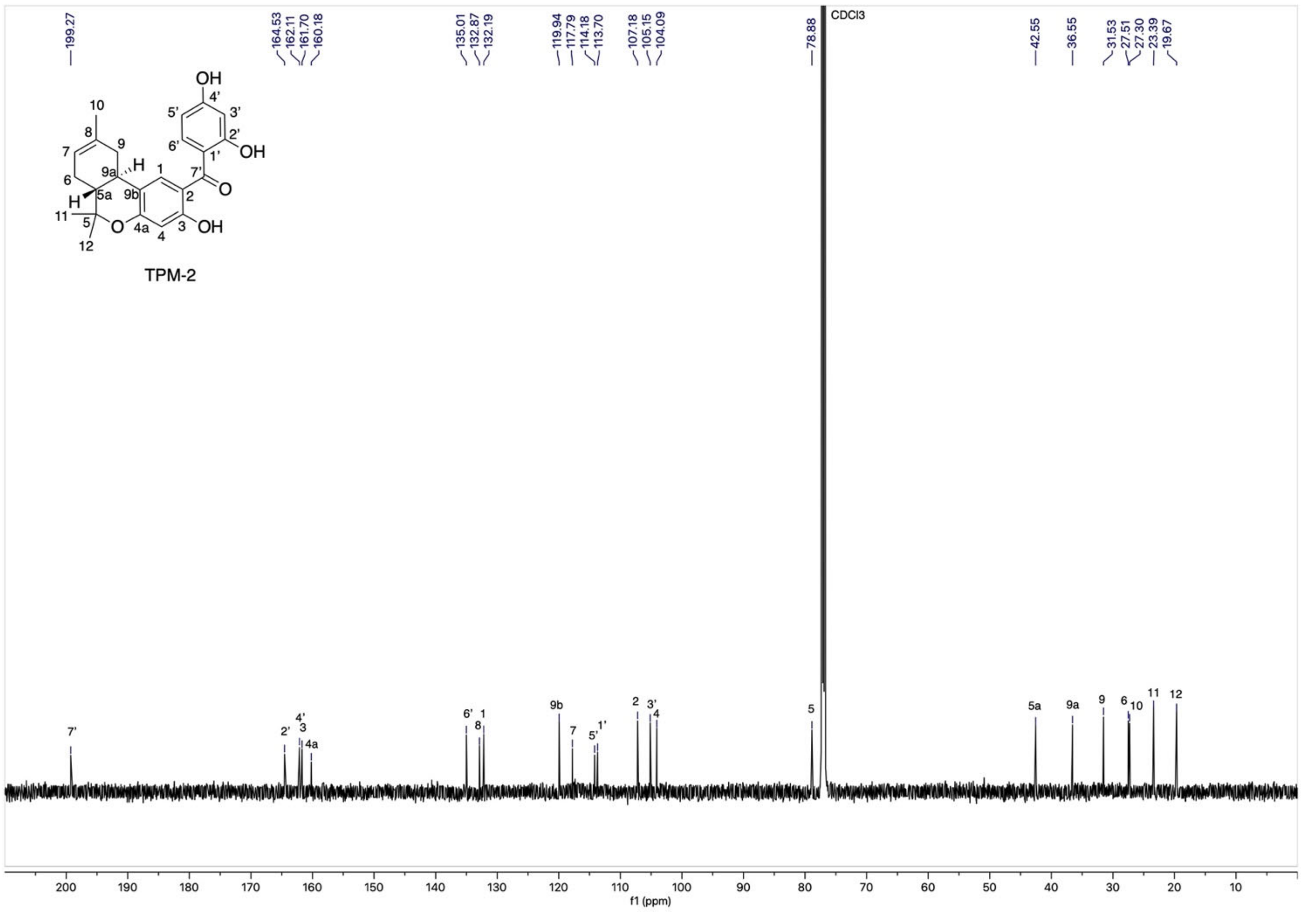

Figure S10. ^{13}C NMR ($CDCl_3$, 150 MHz) of TPM-2

3.4 Annotated ^1H and ^{13}C NMR Spectra for CPM-2

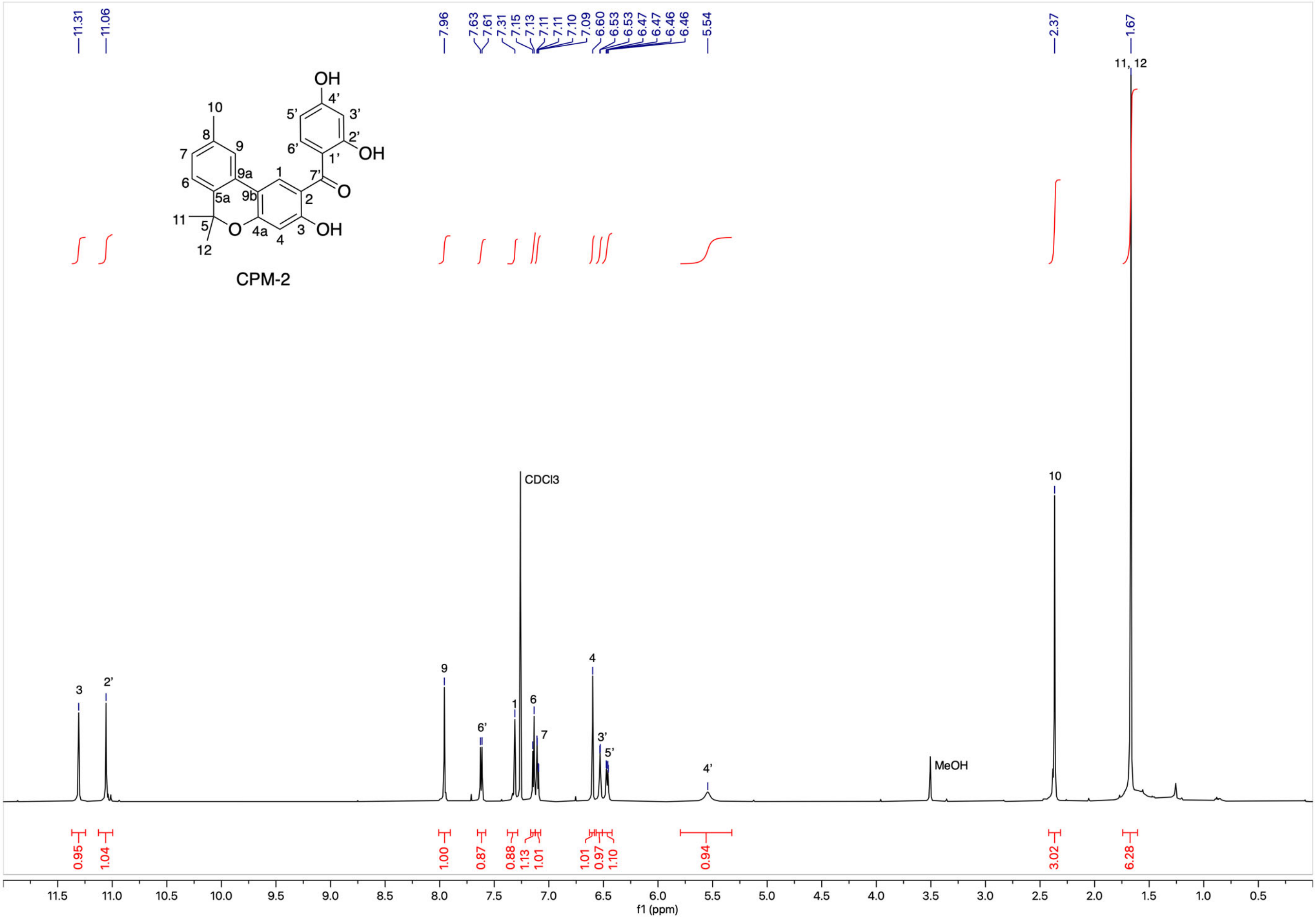

Figure S11. ^1H NMR ($CDCl_3$, 600 MHz) of CPM-2

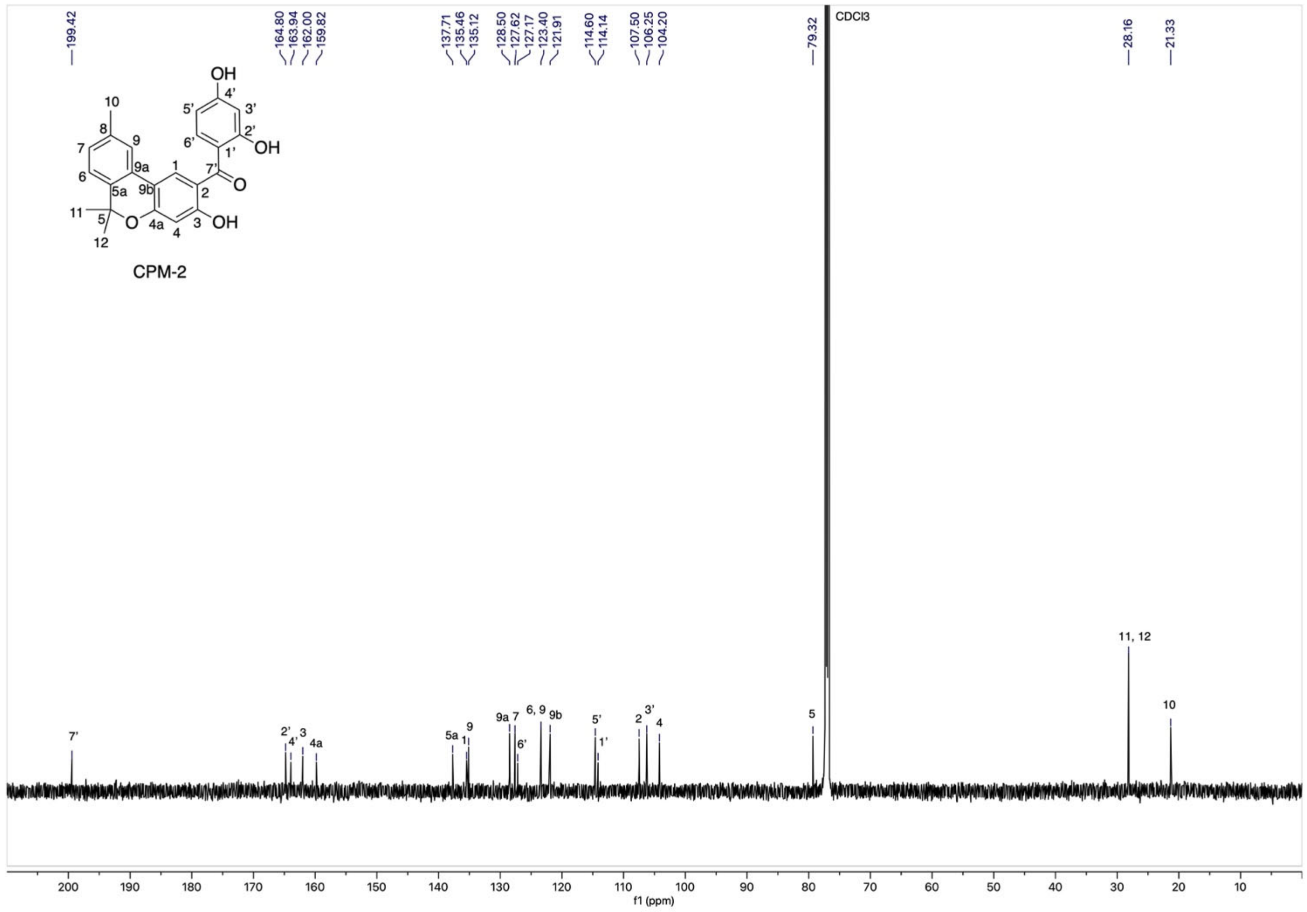

Figure S12. ^{13}C NMR ($CDCl_3$, 150 MHz) of CPM-2

4. HPLC Traces

4.1 HPLC Chromatogram of TPM-1 and TPM-2

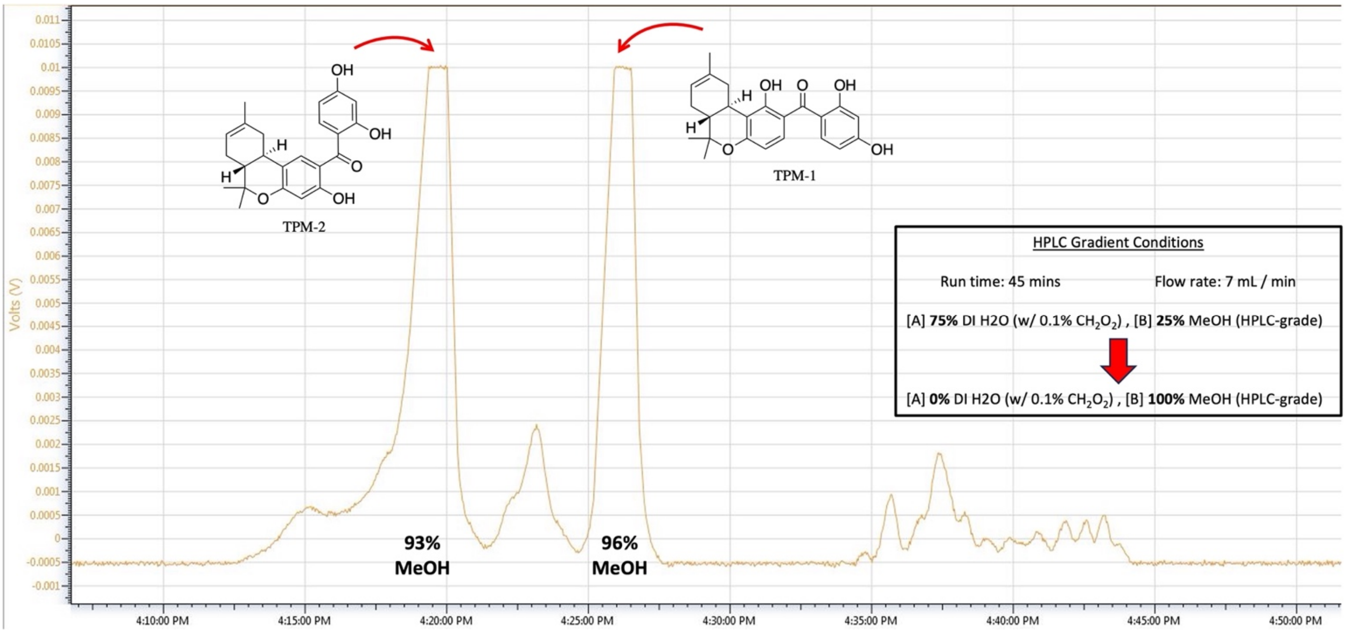

Figure S13. HPLC chromatogram for the isolation of TPM-2 (eluted at 7% H_2O, 93% MeOH) and TPM-1 (eluted at 4% H_2O, 96% MeOH)

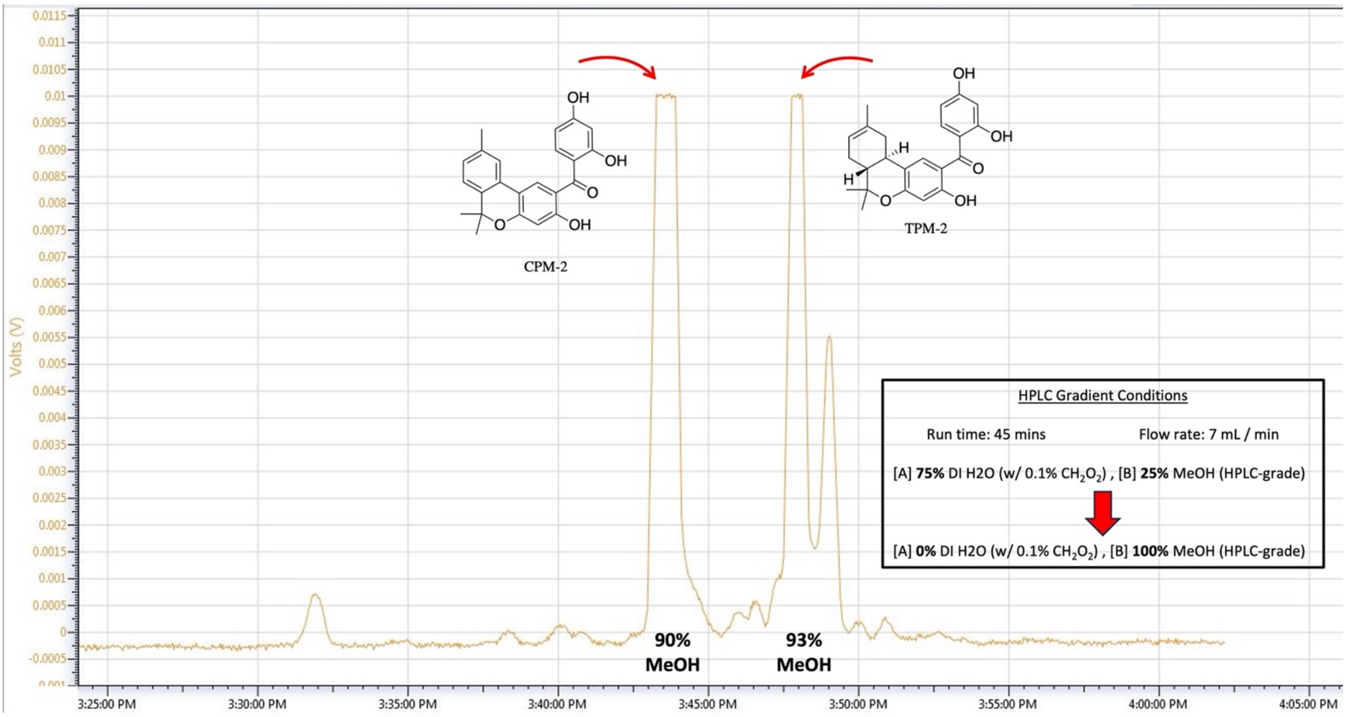

Figure S14. HPLC chromatogram for the isolation of CPM-2 (eluted at 10% H_2O, 90% MeOH) and residual TPM-2 (eluted at 7% H_2O, 93% MeOH).

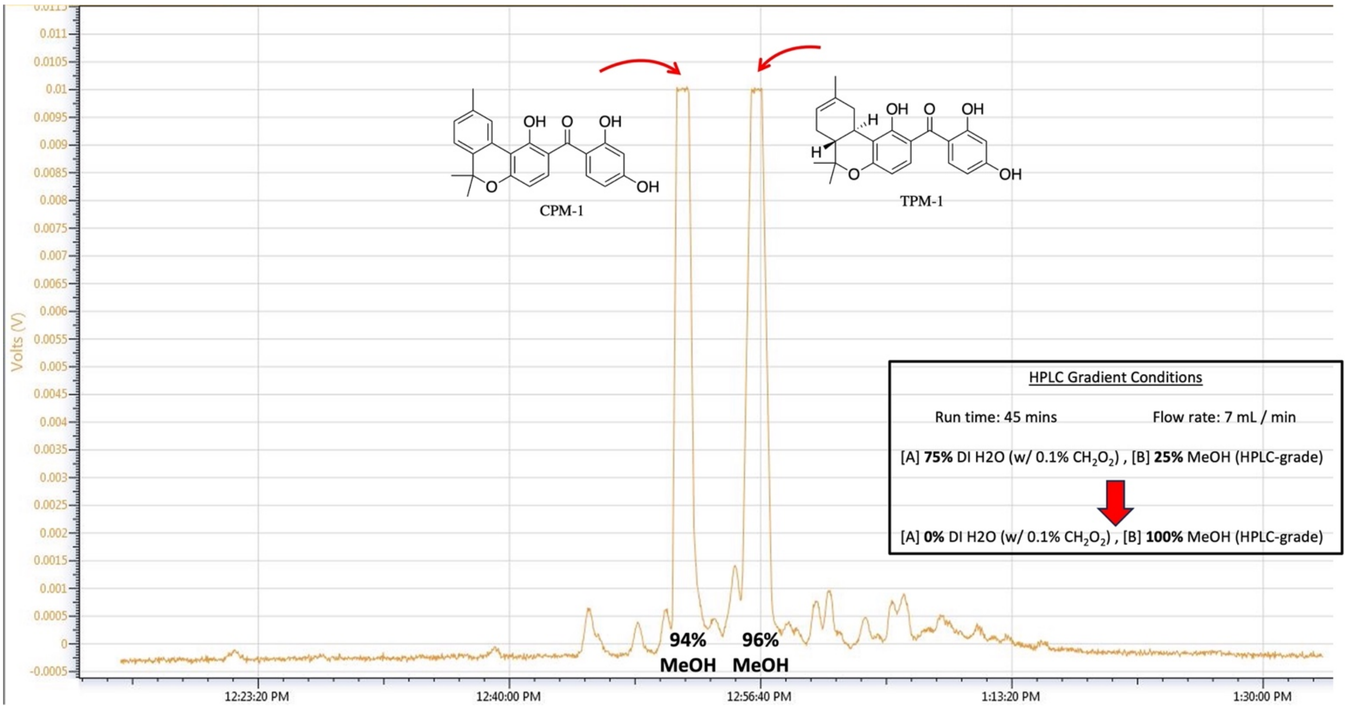

Figure S15. HPLC chromatogram for the isolation of CPM-1 (eluted at 6% H_2O, 94% MeOH) and residual TPM-1 (eluted at 4% H_2O, 96% MeOH).

References

1. Zandi, K.; Amblard, F.; Musall, K.; Downs-Bowen, J.; Kleinbard, R.; Oo, A.; Cao, D.; Liang, B.; Russell, O.O.; McBrayer, T.; et al. Repurposing Nucleoside Analogs for Human Coronaviruses. *Antimicrob. Agents Chemother.* **2020**, *65*, 940.
2. Rosas-Lemus, M.; Minasov, G.; Shuvalova, L.; Inniss, N.L.; Kirryukhiina, O.; Brunzelle, J.; Satchell, K.J.F. High-resolution structures of the SARS-CoV-2 2′-O-methyltransferase reveal strategies for structure-based inhibitor design. *Sci. Signal.* **2020**, *13*, eabe1202.
3. Berman, H.M.; Westbrook, J.; Feng, Z.; Gilliland, G.; Bhat, T.N.; Weissig, H.; Shindyalov, I.N.; Bourne, P.E. The protein data bank. *Nucleic acids research* **2000**, *28*, 235-242.
4. Chen, Y.; Su, C.; Ke, M.; Jin, X.; Xu, L.; Zhang, Z.; Wu, A.; Sun, Y.; Yang, Z.; Tien, P. Biochemical and structural insights into the mechanisms of SARS coronavirus RNA ribose 2′-O-methylation by nsp16/nsp10 protein complex. *PLoS pathogens* **2011**, *7*, e1002294.
5. Trott, O.; Olson, A.J. AutoDock Vina: improving the speed and accuracy of docking with a new scoring function, efficient optimization, and multithreading. *Journal of computational chemistry* **2010**, *31*, 455-461.
6. Wang, M.; Carver, J.J.; Phelan, V.V.; Sanchez, L.M.; Garg, N.; Peng, Y.; Nguyen, D.D.; Watrous, J.; Kapono, C.A.; Luzzatto-Knaan, T.; et al. Sharing and community curation of mass spectrometry data with Global Natural Products Social Molecular Networking. *Nat. Biotechnol.* **2016**, *34*, 828-837.
7. Shannon, P.; Markiel, A.; Ozier, O.; Baliga, N.S.; Wang, J.T.; Ramage, D.; Amin, N.; Schwikowski, B.; Ideker, T. Cytoscape: a software environment for integrated models of biomolecular interaction networks. *Genome Res* **2003**, *13*, 2498-2504.
8. McNaught, A.D.W., A. International Union of Pure and Applied Chemistry (IUPAC) - Compendium of Chemical Terminology. Available online: https://goldbook.iupac.org/ (accessed on 06 October 2024)
